# Revealing Population Heterogeneity in Vesicle-Based Nanomedicines Using Automated, Single Particle Raman Analysis

**DOI:** 10.1021/acsnano.3c02452

**Published:** 2023-06-06

**Authors:** Catherine Saunders, James E. J. Foote, Jonathan P. Wojciechowski, Ana Cammack, Simon V. Pedersen, James J. Doutch, Hanna M. G. Barriga, Margaret N. Holme, Jelle Penders, Mohamed Chami, Adrian Najer, Molly M. Stevens

**Affiliations:** † Department of Materials, Department of Bioengineering, and Institute of Biomedical Engineering, Imperial College London, London SW7 2AZ, United Kingdom; ‡ ISIS Neutron and Muon Source, Rutherford Appleton Laboratory, Science and Technology Facilities Council, Didcot OX11 ODE, United Kingdom; § Department of Medical Biochemistry and Biophysics, Karolinska Institutet, SE-171 77 Stockholm, Sweden; ∥ BioEM Lab, Biozentrum, University of Basel, Mattenstrasse 26, 4058 Basel, Switzerland

**Keywords:** single particle, drug-loading, nanomedicines, polymersomes, Raman spectroscopy

## Abstract

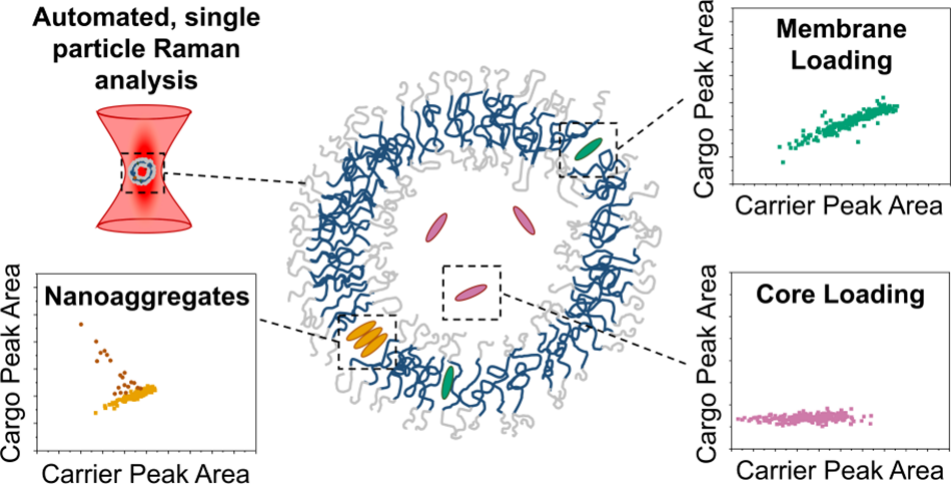

The intrinsic heterogeneity of many nanoformulations is currently challenging to characterize on both the single particle and population level. Therefore, there is great opportunity to develop advanced techniques to describe and understand nanomedicine heterogeneity, which will aid translation to the clinic by informing manufacturing quality control, characterization for regulatory bodies, and connecting nanoformulation properties to clinical outcomes to enable rational design. Here, we present an analytical technique to provide such information, while measuring the nanocarrier and cargo simultaneously with label-free, nondestructive single particle automated Raman trapping analysis (SPARTA). We first synthesized a library of model compounds covering a range of hydrophilicities and providing distinct Raman signals. These compounds were then loaded into model nanovesicles (polymersomes) that can load both hydrophobic and hydrophilic cargo into the membrane or core regions, respectively. Using our analytical framework, we characterized the heterogeneity of the population by correlating the signal per particle from the membrane and cargo. We found that core and membrane loading can be distinguished, and we detected subpopulations of highly loaded particles in certain cases. We then confirmed the suitability of our technique in liposomes, another nanovesicle class, including the commercial formulation Doxil. Our label-free analytical technique precisely determines cargo location alongside loading and release heterogeneity in nanomedicines, which could be instrumental for future quality control, regulatory body protocols, and development of structure–function relationships to bring more nanomedicines to the clinic.

Nanoformulations hold the potential to revolutionize drug delivery due to their improved biostability, biodistribution and targeting potential compared to traditional small molecule drugs.^1−5^*In vivo* nanomedicine behaviors, such as pharmacokinetics and toxicity, are strongly influenced by nanoparticle physiochemical properties, which in turn depend on the underlying heterogeneous nature of the nanoparticles.^6−8^ To date, particle-to-particle heterogeneity is not sufficiently described by standard bulk nanomaterials characterization techniques, such as dynamic light scattering (DLS) or high performance liquid chromatography (HPLC).^9^ Meanwhile many single particle techniques, such as electron microscopy, are low throughput so cannot measure enough particles to give representative population-level information, and visualization of the drug cargo is often challenging. This lack of tools to understand nanoformulation heterogeneity hinders nanoformulation translation to the clinic for several reasons. First, manufacturing processes must be robust and scalable to control particle heterogeneity, with reliable quality control characterization to monitor nanoformulation properties.^1,10^ Second, regulatory bodies must define critical quality attributes describing the acceptable ranges of nanoformulation properties to ensure product quality.^1,10,11^ Finally, a lack of understanding of nanoformulation heterogeneity prevents the development of structure–function relationships to allow the rational design of future nanoformulations.

Despite these challenges, there are 14 FDA approved nanoformulations based on liposomes, which consist of a self-assembled lipid membrane enclosing an aqueous core, due to their biocompatibility and biodegradability.^12,13^ However, their structural analogues prepared from amphiphilic block copolymers, polymersomes, have not yet been translated, despite offering more tunable physical properties and mechanical strength.^14,15^ Both these nanovesicle structures offer two chemically distinct regions within the same particle, which can be flexibly loaded with cargoes of different hydrophilicities simultaneously: more hydrophilic compounds load into the core while hydrophobic compounds partition into the membrane.^16−18^ It is essential to understand cargo chemistry and loading location, given the influence of these parameters on drug leakage and nanocarrier stability.^14,17,19,20^ These factors control nanoformulation stability *in vivo* and cargo release profile; however, it is not standard practice to characterize cargo loading location in vesicles in high throughput.

It is therefore essential to develop methods to study nanocarrier and drug cargo simultaneously on a single particle degree at the population level to promote the development and translation of nanoformulations. This need for additional analysis techniques for detailed nanomedicine characterization has led to the recent development of several high-throughput, single particle techniques to study drug loading in nanoformulations.^9^ Fluorescence-based techniques, such as convex lens-induced confinement (CLiC) and fluorescence correlation spectroscopy (FCS) offer high sensitivity for quantitative loading information for dyes, siRNA and proteins.^21,22^ Additionally, nanoflow cytometry (nano-FCM) can detect loading behavior of fluorescent cargoes such as nucleic acids and doxorubicin.^9^ However, these fluorescence-dependent techniques require the optimization of labeling strategies or inherently fluorescent cargoes. Furthermore, labeling small molecules significantly changes their physicochemical properties, because the dye labels make up a large fraction of the overall molecule. Hence, partitioning and heterogeneity within the nanomedicine and their influence on downstream effects such as stability and release cannot be determined accurately.

An alternative technique that can record label-free, nondestructive, native-state chemical information is Raman spectroscopy. Raman spectroscopy has been used to study cargo loading in both lipid and polymer-based nanomedicines.^23,24^ In particular, Raman spectroscopy can be combined with optical trapping, where a single particle is stably held in the confocal volume of a focused laser beam and its Raman spectrum is simultaneously recorded. After laser shuttering, the particle diffuses away and another particle is trapped in the confocal volume. Previously, this trapping and particle release had been done manually, leading to a low throughput of typically <10 particles per sample.^25,26^ However, the recently developed single particle automated Raman trapping analysis (SPARTA) platform has automated this process to allow the measurement of a few hundred particles per hour.^27^ This high-throughput nature of SPARTA improves the reliability of the data and offers representative population data at the single particle level. Since its establishment, the SPARTA technique has been used for composition analysis of liposomes and polymersomes,^27−29^ reaction monitoring of nanoparticle functionalization^27^ and determining nanoparticle permeability.^30^ However, it has not yet been used for detailed characterization of nanomedicine loading heterogeneity or cargo loading location.

We present here a technique to distinguish cargo location and loading heterogeneity in nanovesicle structures, utilizing high-throughput, single particle Raman spectroscopy data from SPARTA. We first designed and synthesized a library of model cargoes with similar chemical structure but increasing hydrophilicity. We loaded these cargoes in increasing feed amounts into model polymersomes prepared from poly(2-methyl-2-oxazoline)-*b*-poly(dimethylsiloxane)-*b*-poly(2-methyl-2-oxazoline) (PMOXA-*b*-PDMS-*b*-PMOXA). We then measured these loaded polymersomes with SPARTA to obtain label-free, high-throughput information about the polymeric carrier and model cargo on a single particle level. We submitted these data to a comprehensive analysis method including data dimensionality reduction, linear analysis, and non-negative matrix factorization (NNMF). We used this analysis to model the impact of cargo chemistry on loading location and heterogeneity in nanovesicles, supported by Raman imaging of giant polymersomes, electron microscopy, neutron scattering, and simple theoretical modeling. We then applied this model to additional systems, including our model cargoes loaded into liposomes, chloroquine (CQ)-loaded liposomes, and the commercial lipid-based chemotherapy Doxil, confirming broad applicability of this method. We finally used our analysis framework to compare stability and release behavior of the commercial Doxil system and an intentionally leakier formulation, to demonstrate the usefulness of our method when applied to clinically relevant questions.

## Results and Discussion

### Model Cargo-Carrier System Design and Development

**Scheme 1:**
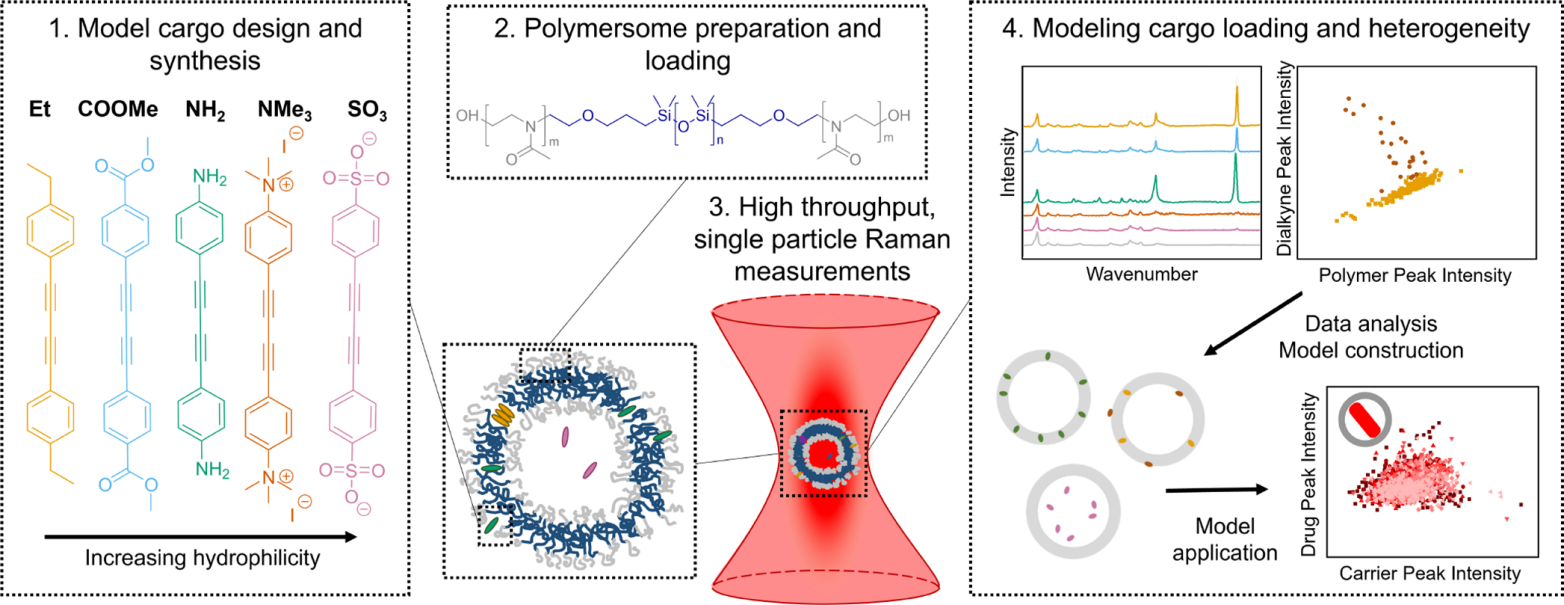
Modeling Cargo Loading Location and Heterogeneity Using SPARTA Measurements Model cargo library with strong, distinct Raman signals and varying hydrophilicity was synthesised by Glaser-Hay coupling. PMOXA-*b*-PDMS-*b*-PMOXA polymersomes loaded with model cargoes were prepared from using a thin-film rehydration method, then measured with SPARTA. SPARTA data was analyzed via dimensionality reduction, linear analysis and NNMF to build physical model of cargo loading. This model was then applied to describe cargo loading in liposomes, including the commercial nanoformulation Doxil.

To create a model for systematic analysis of cargo loading location and heterogeneity, we first designed a series of small molecule model cargoes with increasing hydrophilicity and distinct Raman peaks (Scheme 1). All model cargo molecules were based on the same symmetrical, 1,4-diphenyl-buta-1,3-diyne moiety to reduce the variation of chemical factors such as sterics and π-π stacking, and instead to predominantly focus on the impact of cargo hydrophilicity on loading behavior. Symmetrical dialkynes have a strong, distinct symmetrical C≡C stretching vibrational mode in the Raman silent region,^31−33^ which improves the sensitivity and easily distinguishes cargo from carrier in Raman spectra. The inclusion of phenyl-capping groups improved chemical stability and further increased Raman signal.^34^ We tuned the hydrophilicity by modifying the *para* substituent of the phenyl groups. The 5-member library was then synthesized using Glaser-Hay couplings, a Cu-catalyzed homocoupling of terminal alkynes, which is well established for dialkyne synthesis (Supporting Information).^35−38^ Successful synthesis and purification were confirmed using ^1^H and ^13^C NMR, mass spectrometry (MS) and Raman spectroscopy.

We subsequently loaded these model cargo compounds into polymersomes formed from the amphiphilic triblock copolymer PMOXA-*b*-PDMS-*b*-PMOXA. This polymer type represents one of the most studied polymeric vesicle systems due to its biostability and biocompatibility, which has encouraged its development for nanoreactors, artificial organelles and cell-like entities at the micron-scale, as well as for delivery of small molecules, proteins/enzymes, nucleic acids, and for the formulation of nanoscale pathogen inhibitors.^16,28,39−41^ We selected a commercially available copolymer with 11-65-11 MOXA-DMS-MOXA repeating units. This ratio of hydrophilic:hydrophobic units favors the formation of polymersomes over other possible structures such as micelles and worms.^15,28^ We prepared polymersomes with 0.1, 0.5, 1, 1.5, and 2 mM initial feed of each model cargo using thin film rehydration and extrusion. The hydrophobic cargoes **Et**, **COOMe** and **NH**_**2**_ were dried into a film with PMOXA-*b*-PDMS-*b*-PMOXA, which was then rehydrated with Dulbecco’s phosphate buffered saline (DPBS). The hydrophilic cargoes **NMe**_**3**_ and **SO**_**3**_ were dissolved in DPBS, which was used to rehydrate a film of polymer only. After overnight stirring and extrusion through a 100 nm pore size membrane, the loaded polymersomes were separated from free cargo by size exclusion chromatography (SEC).

**Figure 1:**
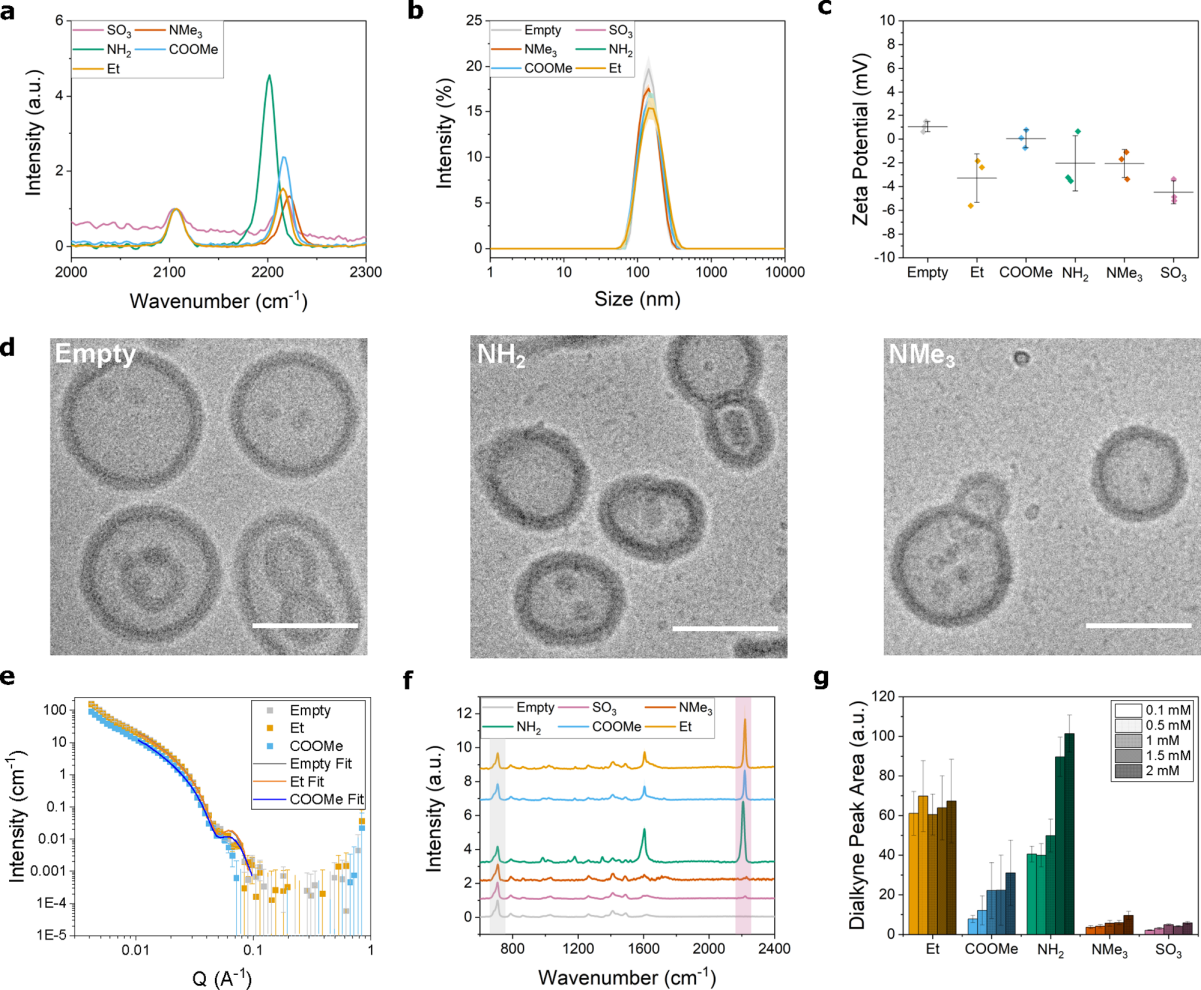
Characterization of polymersome morphology and chemistry. (a) Relative Raman intensity of each model cargo in DMSO at 2 mM (peak at 2208–2225 cm^–1^) compared to EdU at 40 mM (peak at 2107 cm^–1^). One representative repeat shown in main with additional repeats in Supporting Figure S1. (b) DLS intensity distributions of polymersomes in DPBS loaded with 2 mM of each model cargo (mean ± s.d., *n* = 3 technical repeats). (c) Average ζ potential in 300 mM sucrose of polymersomes prepared with 2 mM of each cargo (mean ± s.d., *n* = 3 technical repeats). (d) Cryo-TEM images of unloaded and polymersomes loaded with 2 mM **NH**_**2**_ cargo and 2 mM **NMe**_**3**_ cargo. Scale bars 100 nm. (e) SANS curves of empty, **Et** and **COOMe**-loaded polymersomes. Raw data are represented as points, and fits are shown as lines (fit parameters in Table S1). (f) Mean single particle spectra of polymersomes loaded with 2 mM of each model cargo, measured with SPARTA and normalized to C–Si PDMS peak at 708 cm^–1^ (*n* > 180, mean ± s.d.). Shaded areas highlight the polymer C–Si peak at 708 cm^–1^ (gray) and the cargo C≡C–C≡C peak at 2220 cm^–1^(pink). (g) Dialkyne peak area from spectra normalized to 708 cm^–1^ peak for polymersomes loaded with increasing amounts of each model cargo from SPARTA data. Mean ± s.d. across each particle population, *n* > 180.

To characterize particle properties, we employed dynamic light scattering (DLS), ζ potential and cryogenic transmission electron microscopy (cryo-TEM). DLS revealed that polymersomes were monodisperse with a polydispersity index (PDI) < 0.1 and a similar average size of ∼150 nm (Figure 1b) across loading with all model cargoes at all feed amounts. The ζ potential for polymersomes loaded with all cargoes (Figure 1c) was neutral (−4.5–0.3 mV) which indicates a neutral particle surface charge, as previously reported for PMOXA-*b*-PDMS-*b*-PMOXA polymersomes.^28^ Cryo-TEM images of empty polymersomes showed predominantly vesicle structures, which were conserved when particles were loaded with **NH**_**2**_ and **NMe**_**3**_ (Figure 1d). This is in agreement with a previous study where loading hydrophobic compounds into polymersomes did not change vesicle morphology.^42^ From cryo-TEM, the vesicles had an average membrane thickness of 13.7 ± 1.7 nm (further images in Supporting Figure S2). While cryo-TEM can reveal detailed single particle morphological features, we further used small angle neutron scattering (SANS) to characterize the bulk-level particle size and membrane thickness of empty, **Et** and **COOMe**-loaded particles (Figure 1e). We used SASView v5.0.4 (https://www.sasview.org/) to fit SANS curves with a core–shell ellipsoid model which gave a radius of 83–86 nm and membrane thickness of 14–15 nm for all samples, confirming vesicle morphology and indicating negligible changes after cargo loading, in agreement with cryo-TEM and DLS.

After confirming that we had prepared predominantly monodisperse polymersomes, we characterized particle chemistry and loading using SPARTA. SPARTA is a high-throughput, single particle technique based on Raman trapping which simultaneously measures carrier and cargo chemistry.^27^ In a typical measurement, around 200–300 particles were individually optically trapped, while their particular Raman spectrum was simultaneously recorded. The mean Raman spectrum across all measured polymersomes loaded with 2 mM **Et** (Figure 1f, mean of *n* > 180 traps per sample) shows strong signals from both the polymer and the cargo. Detailed peak assignments are shown in Table 1.^28,31,43^ The main nanocarrier polymer peaks include the Si–C stretch from the hydrophobic PDMS block at 708 cm^–1^ and overlapping CH_2_/CH_3_ deformations at 1411–1437 cm^–1^. Peaks from the **Et** model cargo include the aromatic ring at 1601 cm^–1^ and the main dialkyne vibration at 2220 cm^–1^, as previously reported for similar molecules.^31,34^ Notably, the dialkyne peak position for **NH**_**2**_ is slightly lower, at 2208 cm^–1^, likely due to delocalization from the nitrogen lone pairs into the dialkyne π-bond system, lowering the bond energy and reducing Raman shift.^31^ The presence of characteristic polymer peaks together with similar peaks present for all cargoes implies that each cargo was successfully loaded into polymersomes. Noticeably, the dialkyne peak intensity is not strongly correlated to cargo hydrophobicity. To further investigate cargo loading behavior, we examined Raman peak area to deduce relative amounts of model cargoes upon increasing initial feed amount (Figure 1g).

**Table 1:**
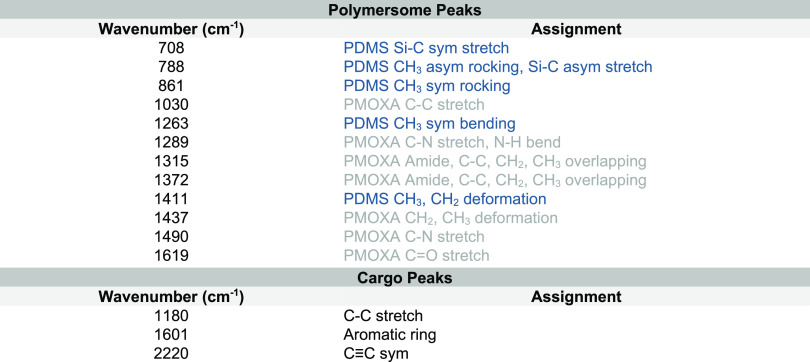
Dialkyne and Polymer Raman Peak Assignments^a^

a PMOXA peaks are shown in gray, PDMS peaks in blue, and cargo peaks in black.^28,31,43^

The impact of initial cargo loading amount on the area of the dialkyne peak at 2220/2208 cm^–1^ (Figure 1g) reveals distinct loading behavior between the different model cargoes. The dialkyne peak area for cargo **Et** was constant at ∼60 across all feed amounts. The hydrophobic ethyl substituent on **Et** suggests a strong partitioning into the membrane. We propose two possible explanations for the consistent loading amount of **Et** despite the increasing initial loading amount. First, it may be that due to the high hydrophobicity of **Et**, polymer-cargo interactions were not as favorable as cargo-cargo interactions, meaning the polymer membrane was saturated below 0.1 mM feed amount, and excess **Et** precipitated out and was removed during purification. Second, the kinetics of particle self-assembly may have been slower than cargo precipitation above 0.1 mM feed in the thin film, with the same result that excess cargo precipitated and did not load into the polymersomes. Meanwhile, the less hydrophobic compounds **COOMe** and **NH**_**2**_ both showed dialkyne peak areas sequentially increasing with feed from 8 to 31 and 40 to 101, respectively (Figure 1g). While these cargoes are also expected to show partitioning into the membrane, the increase in **COOMe** and **NH**_**2**_ Raman signal with feed amount indicates that their lower hydrophobicity induces more favorable interactions with PDMS. This could cause the observed higher loading with increasing feed ratio.

The two more hydrophilic cargoes **NMe**_**3**_ and **SO**_**3**_ also showed increased dialkyne peak area with increasing initial cargo amount, but at 2 to 10, this was at much lower scale than the other cargoes (Figure 1g). These differences are likely due to the difference in cargo chemistry influencing the loading method and location. Due to their hydrophilicity, **NMe**_**3**_ and **SO**_**3**_ are expected to load primarily into the polymersome core. The passive loading of hydrophilic cargo into the aqueous core of nanovesicles is known to be associated with lower encapsulation efficiency than loading hydrophobic cargo into the membrane, since the latter will load actively due to unfavorable interactions with water. This explains the much lower dialkyne peak area for **NMe**_**3**_ and **SO**_**3**_ compared to the other cargoes for the same initial loading ratio. However, the sequentially increasing peak area with increasing cargo feed validates that these hydrophilic cargoes were still loaded into the polymersomes. While examining the mean Raman spectra from SPARTA already reveals differences in cargo loading based on distinct chemistry, the true benefit of SPARTA lies in the ability to measure single particle data. Therefore, we next analyzed single particle-level information in further detail by correlating signal per particle of nanocarrier and cargo.

### Population Analysis of Single Particles Allows Distinction between Membrane and Core Loading

**Figure 2:**
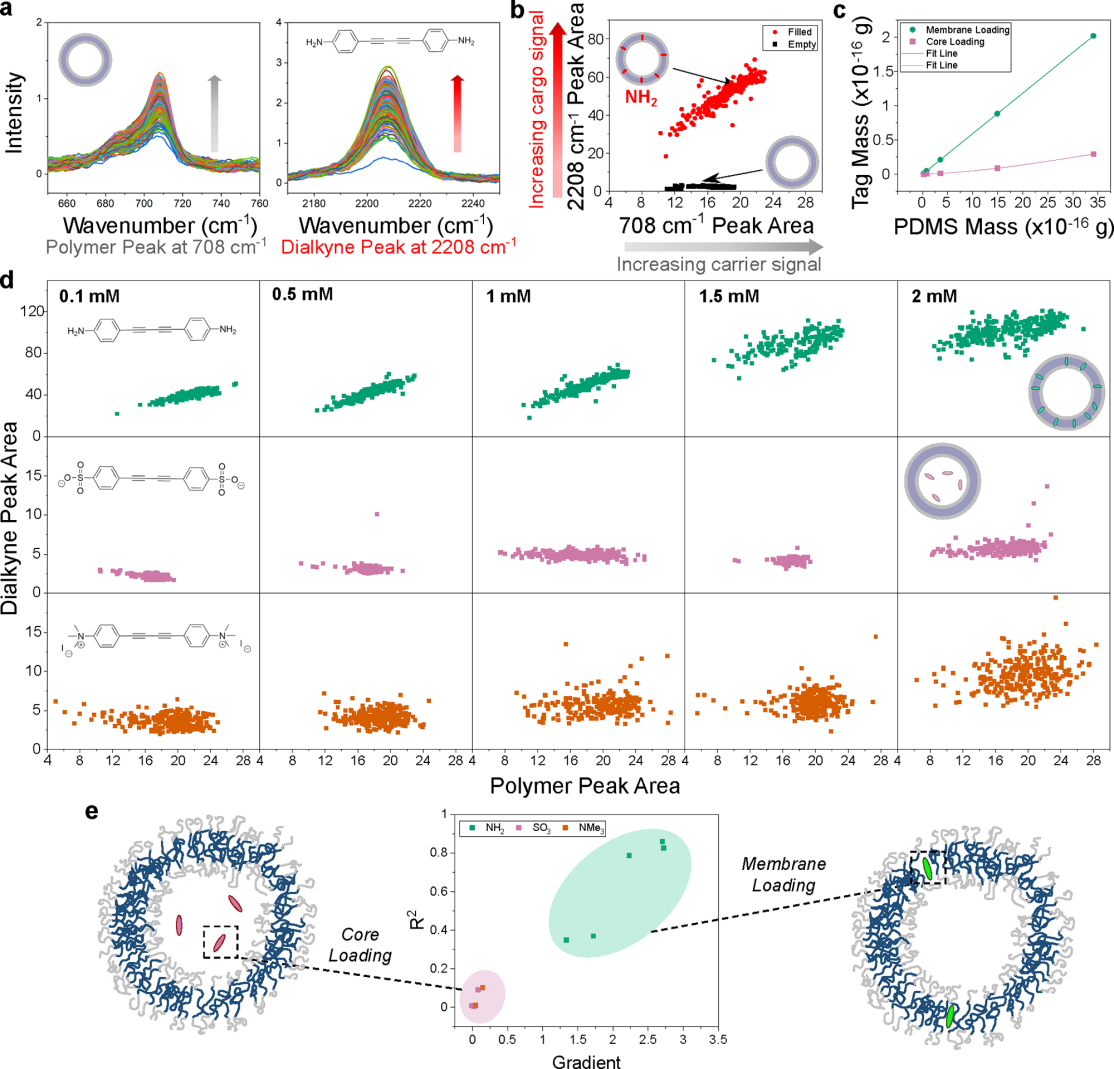
Distinguishing cargo loading location using single particle measurements from SPARTA data. (a) Overlay of all polymer (708 cm^–1^) and cargo (2208 cm^–1^) peaks from one representative SPARTA measurement of an **NH**_**2**_-loaded sample, where each spectra represents one particle trap. These peak areas were used to construct scatter plots showing the relationship between polymer and cargo amount per particle in (b). Also shown in (b) is a scatter plot of an empty sample for reference. (c) Results from theoretical modeling of the relationship between PDMS mass and cargo mass for core and membrane loading cases. Full explanation in Supporting Information. (d) Single-particle scatter plots of polymer peak area against dialkyne peak area for increasing initial loading of 0.1 mM, 0.5 mM, 1 mM, 1.5 mM and 2 mM **NH**_**2**_, **SO**_**3**_ and **NMe**_**3**_, demonstrating the change in cargo loading with amount of polymer. Additional repeats are in Supporting Figure S3. (e) Summary of analytical framework. By extracting the *R*^2^ and gradient from linear fits of cargo-carrier scatter plots, membrane and core loading can be distinguished: core loading causes low gradient and low *R*^2^, so particles appear in the lower left quadrant. Meanwhile membrane loading is characterized by high *R*^2^ and high gradient, so particles appear in the upper right quadrant. Ovals are drawn to aid interpretation.

SPARTA data presents a large, multidimensional data set, consisting of Raman intensities across a ∼1800 cm^–1^ range for ∼200 individual particles for 5 model cargoes at 5 feed amounts. Therefore, to improve the interpretability, we introduced an analytical framework including data reduction. Raman data reduction has previously been achieved by multivariate techniques such as principle component analysis (PCA) and partial least-squares (PLS).^44,45^ However, we can utilize the strong nonoverlapping 708 cm^–1^ PDMS peak (polymersome main signal) and the 2220 cm^–1^ dialkyne peak (cargo main signal) observed in each particle spectrum (Figure 2a). We can then create scatter plots where each point represents the peak areas of one single particle (Figure 2b). Due to the nature of Raman spectroscopy, increasing Raman intensity corresponds broadly to increasing amount of an analyte. Hence, the distribution along the *x*-axis in the scatter plot (Figure 2b) corresponds to variations in membrane amount per particle due to increased particle diameter, multilamellarity, or vesicle-in-vesicle structures, as observed in the cryo-TEM images (Figure 1d). Meanwhile, the *y*-axis distribution describes the relative cargo amount in each single particle. Therefore, our scatter plots allow us to visualize the variation in cargo amount with polymer amount for each of the ∼300 single particles. For an unloaded sample, all particle spectra show a flat distribution at the cargo main signal area (2208/2220 cm^–1^) (Figure 2b). However, a sample loaded with, e.g., 1 mM **NH**_**2**_ shows a strong, positive linear correlation between the polymer and cargo peak areas (Figure 2b). Therefore, particles containing a higher amount of polymer membrane loaded higher amounts of cargo per particle in the case of this hydrophobic cargo.

To understand the data from the scatter plots, we performed simple theoretical modeling to predict the relationship between nanocarrier and cargo masses in the cases of core and membrane loading (Figure 2c, full description in Supporting Information). This corresponds to the scatter plots of 708 cm^–1^ PDMS peak area (carrier) vs 2220 cm^–1^ dialkyne peak area (cargo) from the SPARTA data because the Raman signal is broadly correlated to analyte amount. When cargo is loaded during particle formation, hydrophobic and hydrophilic cargoes display distinct loading behaviors. It is energetically unfavorable for highly hydrophobic molecules to be solubilized in the buffer, so they show strong partitioning into the membrane, which can lead to encapsulation efficiencies of close to 100%. To capture this loading behavior of hydrophobic cargo, we calculated the expected membrane mass for unilamellar vesicles with increasing radius from 25 to 300 nm and used the experimental polymer:dialkyne feed mass ratio to calculate the expected amount of hydrophobic cargo per particle. On the other hand, hydrophilic cargoes reside predominantly in the buffer, so their loading efficiency is limited by the volume entrapped within their nanovesicle core. Therefore, to describe core loading, we calculated the core volume for nanovesicles with increasing radius and used the experimental concentration to calculate resulting encapsulated cargo mass. We have modeled behavior in vesicle structures based on cryo-TEM and SANS data which demonstrated that vesicle structures were preserved upon cargo loading. This simple model required three main assumptions: First, only unilamellar vesicles were present; second, cargoes partitioned completely into either the membrane or the core; and third, there was only even loading rather than nanodomain/nanoaggregate formation.

The modeling results show a positive, linear correlation between PDMS and cargo mass for membrane loading and a much lower, cubic relationship for core loading (Figure 2c). This correlates well with the experimentally observed scatter plots for polymersomes loaded with increasing amounts of hydrophobic **NH**_**2**_, from 0.1 to 2 mM (Figure 2d). In contrast, hydrophilic cargoes, **NMe**_**3**_ and **SO**_**3**_, show lower cargo signals in the scatter plots, in addition to a uniform distribution with polymer amount. With increasing initial loading ratio from 0.1 to 2 mM, the flat distribution incrementally increases in cargo peak area from 2 to 6 for **SO**_**3**_ and 4 to 10 for **NMe**_**3**_. This is partially in agreement with the modeling for core loading. However, the predicted cubic relationship between cargo and carrier is not observed experimentally. This may be due to partial partitioning of these cargoes into the membrane. Additionally, the lower signal from these core-loaded polymersomes would be more influenced by noise, although peak area rather than peak intensity was chosen to mitigate this effect. Finally, core-loaded cargoes would be more affected by factors such as multilamellarity and vesicle-in-vesicle structures (see cryo-TEM, Figure 1d), which reduce the amount of core volume available for cargo to load in but would still provide a membrane for hydrophobic cargoes to partition into. Given that **NH**_**2**_ demonstrated negligible solubility in DPBS, while **NMe**_**3**_ and **SO**_**3**_ were soluble in this aqueous buffer, the difference in these polymer-cargo distributions can be connected to the different cargo hydrophilicities suggesting different loading locations, either within the core (hydrophilic cargoes **NMe**_**3**_ and **SO**_**3**_) or membrane (hydrophobic cargo **NH**_**2**_).

After establishing that we could distinguish qualitatively between core and membrane loading from polymer-cargo peak area scatter plots based on the primary cargo loading location (Figure 2d), we introduced a semiquantitative test to distinguish these loading behaviors (Figure 2e). Core-loading cargoes **NMe**_**3**_ and **SO**_**3**_ formed nearly flat distributions in the cargo-carrier scatter plots, meaning that from a linear fit the gradient was below 0.2, and the correlation coefficient, *R*^2^, was below 0.11 for all loading ratios. Conversely, the membrane loaded **NH**_**2**_ cargo formed a positive, linear correlation between polymer and cargo peak areas, yielding gradients of 1.3–2.7 and *R*^2^ of 0.35–0.86. It should be noted that the two higher feed amounts of **NH**_**2**_, 1.5 and 2 mM, formed more disperse populations (Figure 2d), likely because the membrane was becoming saturated, leading to increased loading dispersity and lower *R*^2^. Despite this, plotting the gradient and *R*^2^ for particles loaded with **SO**_**3**_, **NMe**_**3**_ and **NH**_**2**_ at all feed amounts allows clear distinction between core and membrane loading: core loaded particles fall in the lower left quadrant, while membrane loaded particles appear in the upper right quadrant (Figure 2e).

**Figure 3:**
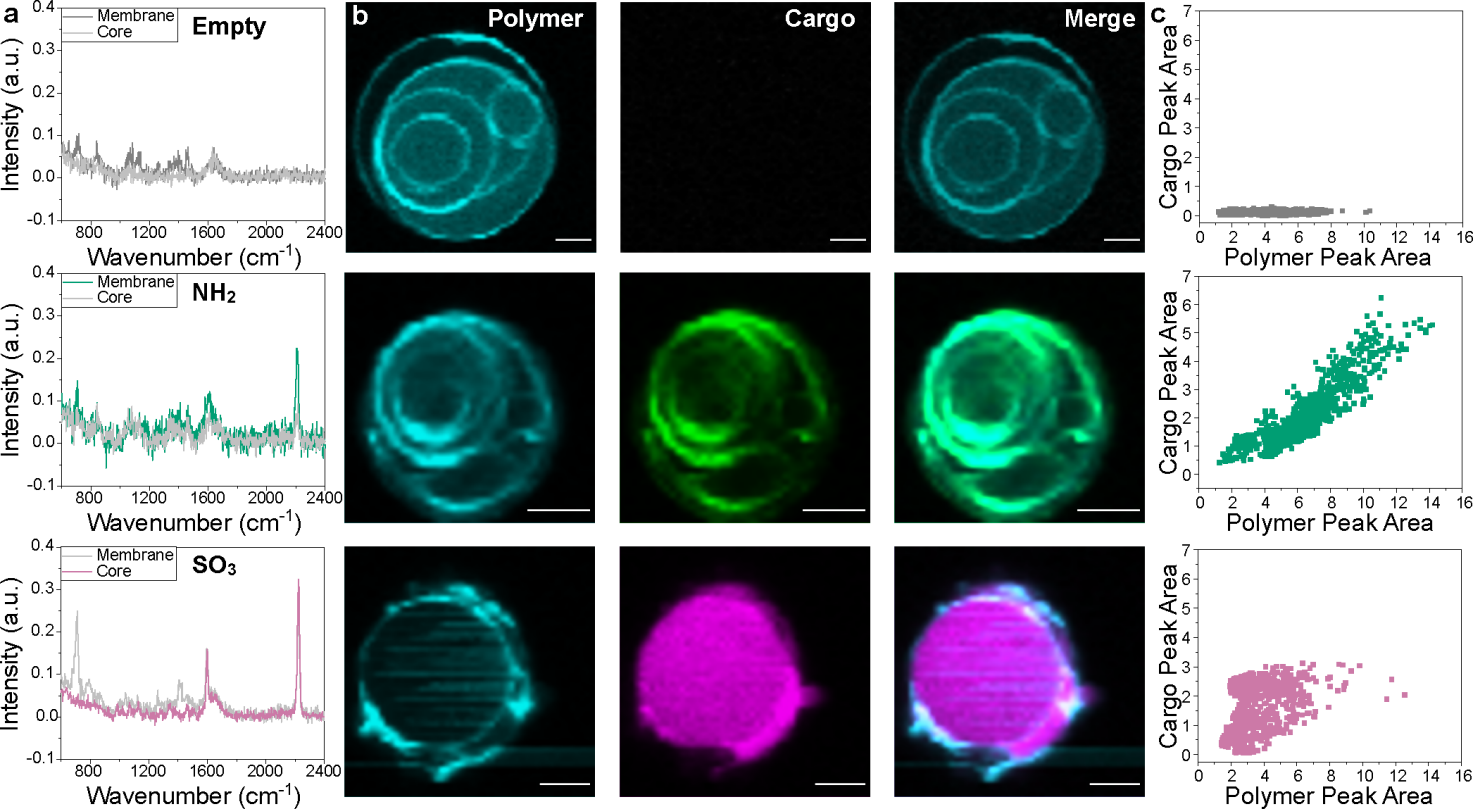
Confocal Raman spectroscopic imaging of giant polymersomes to determine primary loading location of different model cargoes. Confocal Raman imaging was used to visualize loading location of **NH**_**2**_ and **SO**_**3**_ in giant polymersomes, including an empty particle as a control. (a) Example Raman spectra from a single voxel. Two representative voxels from both the membrane and core regions were chosen from each particle loaded with different cargoes. Due to the self-assembly process to form these particles there are varying degrees of multilamellarity between particles. Spectra were normalized to the water peak at 3300 cm^–1^ due to the low intensity of the 708 cm^–1^ polymer peak on this instrumental setup. (b) Univariate peak area analysis was used to reconstruct images of the **NH**_**2**_ signal at 2208 cm^–1^ shown in green and the **SO**_**3**_ signal at 2220 cm^–1^ shown in magenta. Polymer signal, shown in cyan, was determined from 2905 cm^–1^ peak due to lower intensity of 708 cm^–1^ peak with this instrumental setup. Scale bars 5 μm. (c) Scatter plots represent the polymer and dialkyne peak area at each voxel in the Raman images after thresholding to remove voxels containing no particle signal.

To visualize and confirm cargo loading location and validate our method conducted on nanovesicles, we performed 2D confocal Raman imaging on giant polymersomes at the micron scale (Figure 3, additional particles Supporting Figure S4). Giants loaded with **NH**_**2**_ clearly showed colocalization of the dialkyne (cargo) and the polymer membrane. In comparison, hydrophilic **SO**_**3**_ was detected in the giant vesicle core. After thresholding out purely water spectra, we reconstructed scatter plots of the correlation between the polymer and cargo peak areas from these Raman images. However, here each point represents one voxel in the image rather than one particle (Figure 3). As expected, the empty vesicle showed no correlation between the nanocarrier and cargo. The **NH**_**2**_ loaded particle showed a linear correlation between polymer and cargo, meaning that the cargo was colocalized with the membrane within the resolution of the microscope, in agreement with our SPARTA analysis of nanovesicles loaded with the same cargo. Meanwhile most voxels in the **SO**_**3**_ loaded particle had a cargo peak area of ∼2.5, corresponding to the core region. Although voxels containing membrane signals also contained **SO**_**3**_ signals, the confocal volume used for imaging was ∼500 nm in diameter, while the polymer membrane was only ∼14 nm (from cryo-TEM and SANS, Figure 1d,e), meaning that the confocal volume would be filled with both membrane and core volumes. The images confirmed that the hydrophobic **NH**_**2**_ cargo partitioned predominantly into the membrane, while the hydrophilic **SO**_**3**_ partitioned mainly into the core. This confirms the location-dependent differences in scatter plots from the SPARTA data observed previously (Figure 2). With this analytical framework established and an increasing understanding of cargo loading behavior validated through imaging, we examined whether the model cargoes with higher hydrophobicity, **Et** and **COOMe**, behaved similarly.

### Population Heterogeneity Induced by Different Cargoes

**Figure 4:**
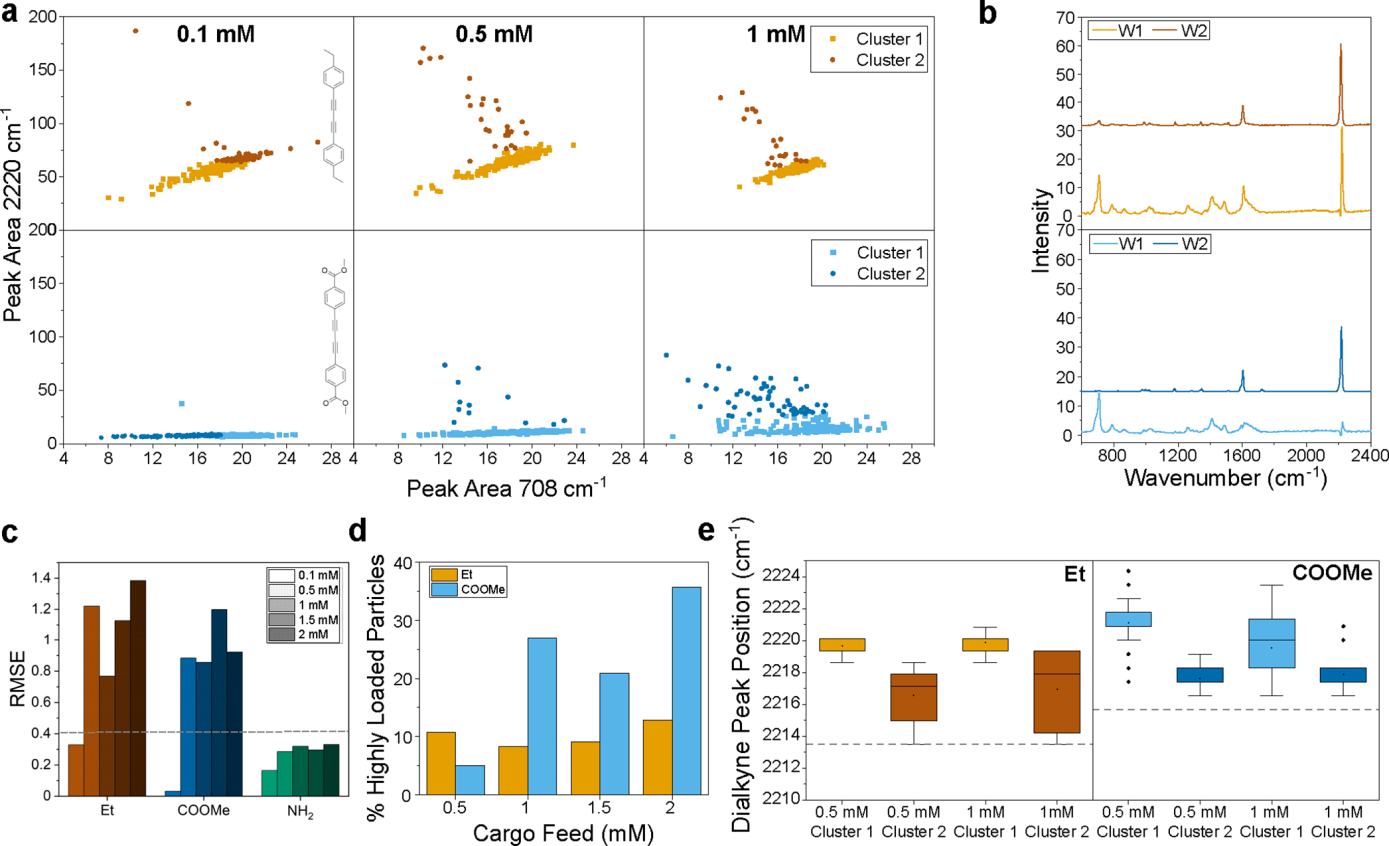
Identifying and quantifying drug loading subpopulations. (a) Scatter plots of 708 cm^–1^ polymer peak area and 2220 cm^–1^ cargo peak area for polymersomes loaded with 0.1 0.5, and 1 mM **Et** or **COOMe**, from SPARTA data. Lighter points indicate particle spectra sorted into cluster 1 by the NNMF clustering method, while darker points indicate particle spectra sorted into cluster 2. (b) Pseudospectra of deconvoluted factors where factor 1 (W1) most closely resembles the polymer spectrum and factor 2 (W2) most closely resembles the cargo spectrum for 1 mM loading in **Et** (top) and **COOMe** (lower). (c) RMSE between dialkyne peaks for all loading ratios of **Et**, **COOMe** and **NH**_**2**_. Dashed line at 0.4 has been used as a visual aid to distinguish between samples with one population (lower) and samples with 2 subpopulations (higher) in terms of cargo loading. (d) Variation of percentage of particle spectra loaded into the outlier cluster 2 by NNMF clustering method with increasing loading ratio for **Et** and **COOMe** cargoes. (e) Box plots summarizing dialkyne peak positions of individual particles in different clusters for polymersomes loaded with 0.5 and 1 mM **Et** (left) and **COOMe** (right) (*n* = 12–231 particles per cluster; center line, median; box limits, interquartile range; whiskers, 1.5 interquartile range values). Dashed lines indicate position of dialkyne peak from powder spectra measurements.

To examine **Et** and **COOMe** loading behavior, polymersomes were loaded with increasing cargo feed from 0.1 to 2 mM **Et** and **COOMe**, similarly to the previous model cargoes, and then measured with SPARTA. The SPARTA scatter plots for particles loaded with **Et** and **COOMe** (Figure 4a) revealed substantially different loading behavior from the model cargoes **SO**_**3**_, **NMe**_**3**_ and **NH**_**2**_ described in the previous section. For all feed amounts, **Et** and **COOMe**-loaded particles formed a main population with a linear correlation between the cargo and polymer, indicating even loading into the membrane, as seen in the membrane-loading **NH**_**2**_ cargo (Figure 2d). However, for loading ratios above 0.5 mM, **Et** and **COOMe** loading also formed a subpopulation of particles with a high amount of cargo relative to the amount of polymer in that particle (Figure 4a). To confirm that this was not specific to the copolymer chosen, we loaded these cargoes into PMOXA-*b*-PDMS-*b*-PMOXA copolymers with different block lengths (Supporting Figure S5) which all formed the same two populations of both a linear loading region and highly loaded particles. These highly loaded particles are an important characteristic of a formulation, since particles with high variability in cargo loading could have different interactions on the cellular level, thus affecting overall nanoformulation behavior. However, these two populations would not be identified with standard characterization techniques such as HPLC or bulk absorbance spectroscopy. We therefore decided to further investigate the subpopulations revealed by SPARTA data.

To quantify these different subpopulations, we employed non-negative matrix factorization (NNMF), which is an unsupervised multivariate technique that factorizes a given matrix into two strictly non-negative matrices. In our case, the given matrix contained the intensities of each individual particle Raman spectrum at each wavenumber, and the deconvoluted factors resembled the empty polymer and model cargo Raman spectra (Figure 4b). In NNMF, each of these factors is given a score per particle based on how much the factor contributes to that spectrum. By assigning each particle to a cluster depending on which factor it scores highest on, particles within a sample can be assigned to distinct clusters, as has previously been applied to analyzing genetic data.^46^ NNMF was run on the whole spectra, which improved the sensitivity, and we then represented the two clusters with our scatter plots of polymer and cargo peak areas.

We ran NNMF with 2 factors separately on each loading ratio for **Et** and **COOMe**. In our NNMF model, factor 1 (W1) resembled the empty polymer spectrum, while factor 2 (W2) resembled the cargo spectrum (Figure 4b). Above 0.5 mM loading, particles sorted into cluster 1 belonged to the linear region, while particles sorted into cluster 2 were the highly loaded particles (Figure 4a). However, at 0.1 mM for both **Et** and **COOMe**, the clustering was based on the amount of polymer, for example, larger or more multilamellar particles, rather than the amount of cargo in a particle, because at this feed amount there were not enough highly loaded particles to form a separate subpopulation (Figure 4a). We therefore designed a simple test to determine whether NNMF could detect separate subpopulations from SPARTA data. After running NNMF, we found the root-mean-square error (RMSE) in the dialkyne peak region (2184–2235 cm^–1^) between the two factors. As a control, we also compared the RMSE values for **NH**_**2**_-loaded particles, which we had previously found to form only one population (Figure 2d). When only one subpopulation was formed, during loading of 0.1 mM **Et** and **COOMe**, and all loading amounts of **NH**_**2**_, the RMSE was below 0.33, whereas the subpopulations were identified by an RMSE above 0.86 (Figure 4c). We quantified the percentage of highly loaded particles where subpopulations were identified, and how this varied with cargo feed (Figure 4d). For **Et**, the proportion of highly loaded particles was stable at 8–13% across all of the feed amounts. For **COOMe**, there was a general increase from 5 to 36% highly loaded particles with increasing cargo feed from 0.1 to 2 mM. Therefore, although average **COOMe** loading was higher than **Et** at higher loading ratios (Figure 1g), there was a larger proportion of highly loaded particles, which would be relevant for examining differences in toxicity for clinical nanoformulations.

In addition to determining the proportion of highly loaded particles, we evaluated their physical origin. DLS and SANS characterizations found no morphological difference between empty polymersomes and polymersomes loaded with **Et** and **COOMe**, which indicates that these particles are highly loaded due to drug distribution rather than significant particle morphology changes (Figure 1b,e). However, there are changes to the dialkyne peak position between the different subpopulations, which indicate changes in the cargo environment. The dialkyne peak position was at 2220 cm^–1^ for particles loaded with **Et** at both 0.5 and 1 mM in the linear loading region, or cluster 1. However, highly loaded particles in cluster 2 demonstrated a lower shift of dialkyne peak position to 2217 cm^–1^ for both loading ratios (Figure 4e). This lower peak position for highly loaded particles is more similar to the powder-state peak position at 2214 cm^–1^. Similarly, **COOMe**-loaded particles in cluster 1 showed average cargo peak positions of 2221 and 2220 cm^–1^ for 0.5 and 1 mM loadings, respectively, while the average dialkyne peak position for the highly loaded subpopulation of particles was at 2218 cm^–1^, which was again closer to the 2216 cm^–1^ peak position recorded for **COOMe** powder (Figure 4e).

The higher dialkyne peak position of cluster 1 particles indicates that cargo molecules in this subpopulation of particles were in a different environment to the lower-wavenumber dialkyne compounds in the highly loaded particle subpopulation. It may be that cluster 1 cargo molecules are predominantly solubilized in the membrane, while highly loaded particles contain nanoaggregates of cargo. This behavior is likely related to the balance of cargo-cargo and cargo-polymer interactions, where the **Et** and **COOMe** cargoes experienced stronger self-interactions, so were more likely to stack than to evenly associate in the polymer membrane. These nanoaggregates were still associated with the particles, as either domains in the membrane or stacks associated with the membrane, as they were measured when trapping a polymer-containing particle. To confirm nanoaggregate formation, we performed Raman imaging of films of PMOXA-*b*-PDMS-*b*-PMOXA with 0.5 mM **NH**_**2**_, **COOMe** and **Et** to test for domain formation (Supporting Figure S6). This confirmed that **NH**_**2**_/polymer films were homogeneous, leading to even loading in the polymersome membrane, while **COOMe**/polymer and **Et**/polymer films showed significant phase separation between polymer and cargo in the films, explaining nanoaggregate formation in the particle samples measured by SPARTA.

Using SPARTA data on our model system, we have developed an analytical method to characterize drug loading at the population level with single particle detail. First, running NNMF clustering followed by RMSE evaluation between the deconvoluted factors can quantify different subpopulations in terms of drug loading. Second, by evaluating the gradient and *R*^2^ of scatter plots of the main carrier and cargo peak areas, we can distinguish core and membrane loading. Our method can therefore offer valuable information on drug loading mechanisms with single particle detail at a population level that is not available with traditional nanoformulation characterization techniques.

### Applying the Model to a Varied Range of Nanovesicle Systems

We then tested whether our model could describe drug loading in nanocarriers beyond polymersomes. We investigated liposomal nanocarriers because there are currently 14 liposome formulations on the market,^13^ so this is a highly relevant nanocarrier. Many approved formulations contain lipids with phosphatidylcholine (PC) headgroups and cholesterol (CH), so we prepared liposomes with dipalmitoylphosphatidylcholine (DPPC):CH in a 4:1 molar ratio. We loaded liposomes with 1 mM **Et**, **NH**_**2**_ and **SO**_**3**_, which exhibited heterogeneous, membrane, and core loading, respectively, in our polymersome model system, to test whether the cargoes would behave similarly in a different nanocarrier. We extruded the liposomes through a 100 nm pore-size membrane, forming particles with average size ∼110 nm and PDI < 0.1 (Supporting Figure S7) so these particles had a similar size distribution to commercial formulations such as Onivyde and Doxil. We then measured the liposomes with SPARTA.

**Figure 5:**
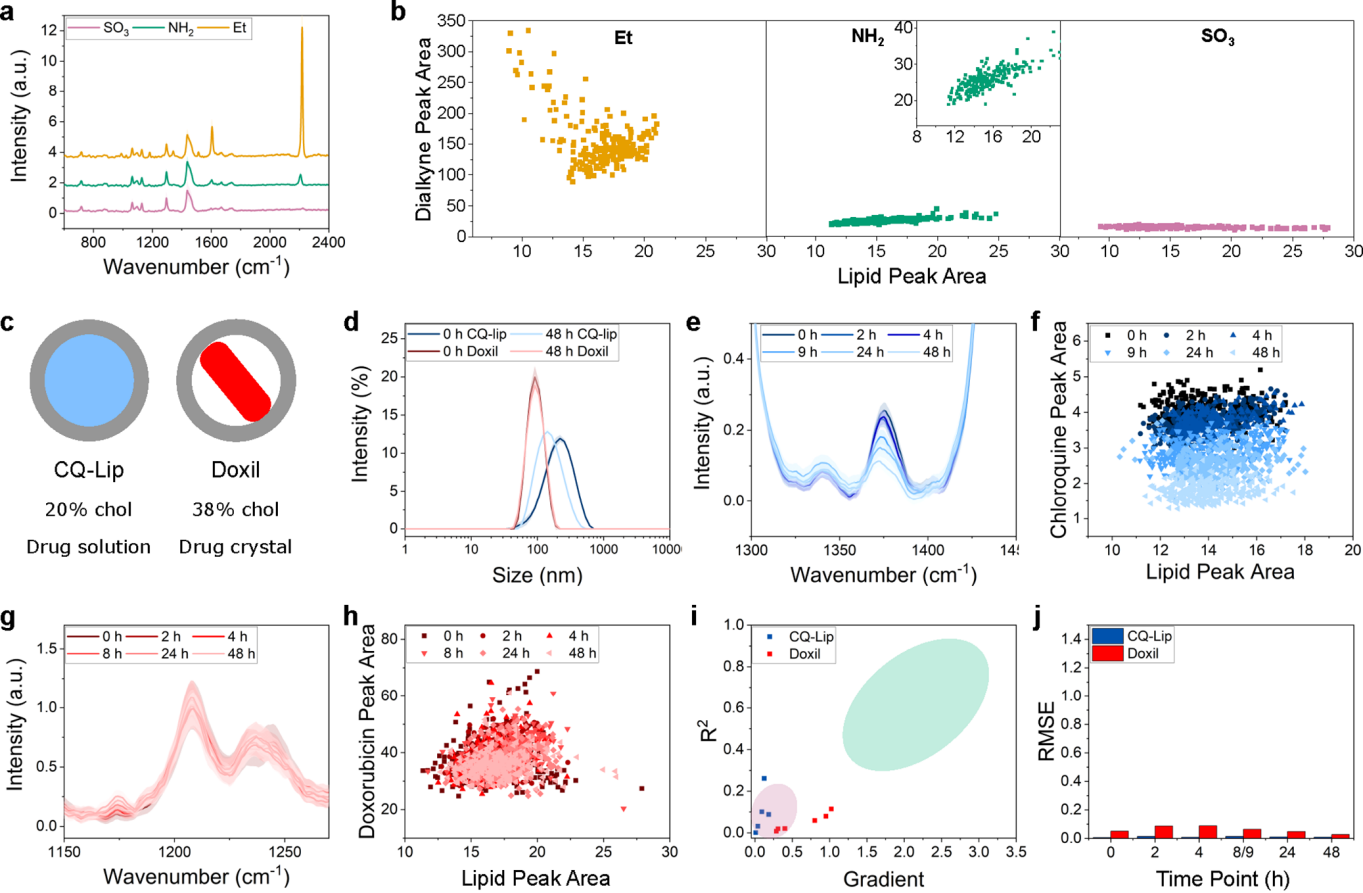
Application of analytical framework to liposomal nanocarriers. (a) Mean Raman spectra ± s.d. across all model cargo-loaded liposomes measured with SPARTA (*n* > 188). (b) Scatter plots of the lipid 1295 cm^–1^ peak area and 2208 (**NH**_**2**_) or 2220 cm^–1^ (**Et** and **SO**_**3**_) dialkyne peak area from SPARTA data. (c) Schematic of chloroquine-loaded liposomes (CQ-lip) and Doxil formulations used for release study. (d) DLS data for CQ-lip and Doxil over 48 h incubation at 37 °C. Mean intensity curves ± s.d. of technical triplicates. Full data set in Supporting Figure S7. (e) Mean Raman spectra ± s.d. across all particles for time points during 48 h incubation for CQ-lip. (f) Scatter plots of 1295 cm^–1^ lipid peak area and 1375 cm^–1^ CQ peak area for all particles at all time points during release study. (g) Mean Raman spectra ± s.d. across all particle traps for Doxil particles measured at various time points during incubation at 37 °C in 10% FBS v/v. (h) Scatter plots of 1295 cm^–1^ lipid nanocarrier peak and 1208/1242 cm^–1^ drug peak area for each particle over 48 h incubation and drug release. (i) Linear analysis of CQ-lip and Doxil scatter plots at all time points sampled during incubation. (g) RMSE evaluation of NNMF clustering for two nanoformulations over 48 h incubation. Scales for (i) and (j) chosen to match with Figures 2e and 4c from previous modeling. Ovals in (i) are repeated from Figure 2e for ease of comparison.

The mean Raman spectra across all particles from SPARTA characterization showed the dialkyne peak at ∼2220 cm^–1^ for **Et** and **SO**_**3**_, or 2208 cm^–1^ for **NH**_**2**_, indicating successful loading. The main lipid peaks included 1295 cm^–1^ CH_2_ twist/wag and 1440 cm^–1^ CH_2_ deformation (full assignment, Table 2).^26,47,48^ Analyzing the SPARTA data within our analytical framework revealed similar loading behavior of the model cargoes in liposomes as previously seen in our polymersome system (Figures 2 and 4). Scatter plots could now be formed using the lipid 1295 cm^–1^ and the dialkyne peak areas. Most particles loaded with **Et** (73% as determined by NNMF, Figure 5b) formed a subpopulation where cargo amount increased linearly with lipid amount. The remaining **Et**-loaded particles formed a highly loaded subpopulation. The RMSE between the two clusters was 2.1, identifying the presence of two subpopulations within our analytical framework. **NH**_**2**_ loaded liposomes formed only one population where cargo and carrier amounts increased linearly with gradient 1.1 and correlation coefficient 0.6, indicating even membrane loading according to our modeling (Figures 5b). **SO**_**3**_-loaded liposomes formed a flat distribution with gradient −0.1 and correlation coefficient 0.2, which suggests core loading according to our model (Figure 5b). Therefore, the model cargoes behaved similarly in liposomes and polymersomes, despite the distinct chemical and mechanical properties of DPPC and PMOXA-*b*-PDMS-*b*-PMOXA. Furthermore, we could validate that our model could be applied to successfully describe loading also in different nanocarrier systems.

**Table 2:**
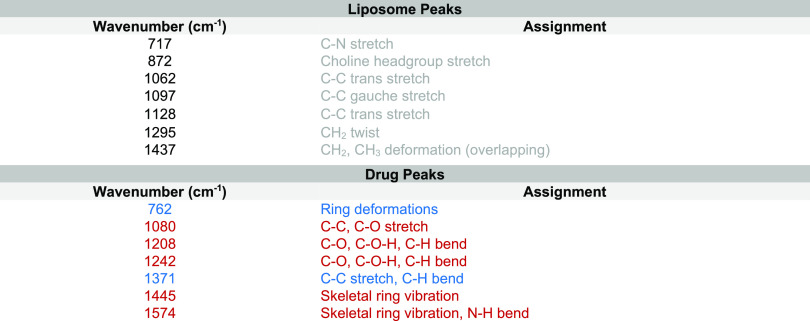
Peak Assignments of Liposomal Nanocarriers Containing Lipids with PC Headgroups and Loaded with Chloroquine or Doxorubicin^a^

a Lipid peak shown in gray, CQ peaks shown in blue, and doxorubicin peaks shown in red.^26,44,48^

We then applied our analytical method to a typical question in nanoformulation development, namely, to test the release of different formulations. We prepared liposomes with lipid composition DPPC:CH 4:1 mol % which were passively loaded with 50 mM chloroquine during particle formation (CQ-lip). We selected this nanoformulation based on previous reports that similar formulations have leaked over a few hours/days, which would allow us to measure the change in drug distribution.^49^ We then dialyzed the formulation for 48 h at 37 °C and measured DLS and SPARTA at various time points. DLS characterization confirmed that over the 48 h period the particles retained a stable size distribution with average diameter 208 nm and PDI below 0.2 (Figure 5d). The mean Raman spectra from SPARTA characterization revealed that the chloroquine peak at 1375 cm^–1^, derived from overlapping C=C, N–H and C–H bonds,^50^ sequentially reduced in intensity over the 48 h incubation from 0.26 to 0.10 (Figure 5e).

We then plotted the correlation between lipid and drug content for each measured particle by plotting the 1295 cm^–1^ peak area against the chloroquine 1375 cm^–1^ peak area for all particles at each time point (Figure 5f), which is analogous to the scatter plots above (Figure 2 and 4). CQ-lip showed low correlation between the lipid carrier and chloroquine cargo, similarly to the core-loaded **SO**_**3**_ and **NMe**_**3**_ model cargoes. Applying our model framework to these scatter plots found gradients from 0.0 to 0.2 and *R*^2^ values below 0.26 (Figure 5i). Additionally, the RMSE at all time points was below 0.02 (Figure 5j). Our previous modeling confirms that the particles were evenly core loaded and released the drug consistently across the population over the 48 h period. This confirms the suitability of our analytical framework for capturing high-throughput, single particle changes in drug distribution during cargo release.

To compare with the CQ-lip release profile and demonstrate applicability to commercial formulations, we studied the release from Doxil, the first FDA-approved liposomal product which is a highly optimized formulation containing the anticancer drug doxorubicin. We tested the ability of SPARTA to measure Doxil in high fetal bovine serum (FBS) concentration (50%, 75% and 90% v:v), as this is more representative of *in vivo* conditions. We could see trapping and particle-associated peaks, even at the highest serum concentration of 90% (v:v) (Supporting Figure S8), which demonstrates the ability of SPARTA to measure nanoformulations in complex environments. This ability could be applied to many research questions, including stability and cargo release testing in relevant conditions. The high background signal from FBS, especially at high percentages, would require additional analysis to deconvolute from the particle spectra, which is beyond the scope of this study. Therefore for release conditions, we dialyzed particles in a lower serum amount with 10% v:v FBS in DPBS (the concentration commonly used in cell culture media), meaning there were still serum components present, but the Raman background signal associated with the serum was now negligible.

Over 48 h of dialysis at 37 °C, Doxil remained stable in size at an average diameter of 97 nm with PDI below 0.1 (Figure 5d). The mean SPARTA spectra across all particles show strong doxorubicin peaks at 1208 and 1242 cm^–1^, from overlapping C–O, C–O–H and C–H bends (Figure 5g). However, in comparison to the CQ-lip, the Doxil peak intensity did not decrease over 48 h. We plotted the 1295 cm^–1^ lipid peak area against the 1208/1242 cm^–1^ doxorubicin peak areas (Figure 5h). Similarly to CQ-lip, the Doxil formulation showed a low gradient (0.3–1.0) and little correlation between nanocarrier and cargo amount (*R*^2^ values 0.0–0.1) (Figure 5i). This is explained by the active loading used for Doxil which causes core loading with the cargo in crystallized form. This shows that variability in doxorubicin crystal size is not strongly correlated to particle membrane amount. RMSE evaluation of NNMF clustering was below 0.09 across all time points, confirming that there was one population throughout the release study (Figure 5j). Overall, our analysis indicated that over 48 h, the drug was mainly contained in the core of Doxil without significant release. This agrees with previous reports that after 48 h Doxil incubation in plasma at 37 °C, only 10% or <5% doxorubicin was released.^51,52^

Our modeling can therefore aid interpretation of formulation heterogeneity and drug release from the two different nanoformulations. Both CQ-lip and Doxil consist of predominantly core-loaded drug with a single population of particles in terms of drug loading. While chloroquine was detectably released from CQ-lip over 48 h, Doxil did not release significant amounts of drug over the incubation period. This is due to the higher cholesterol percentage in the Doxil lipid membranes compared to CQ-lip, 38% compared with 20%, and the crystallized drug in Doxil contrasted with the solubilized drug in CQ-lip. Our method can therefore distinguish between the stability of different nanoformulations by tracking the change in drug distribution on a single particle level over time.

## Conclusions

We have presented an analytical framework using label-free, single particle-based, population level chemical information to distinguish between cargo loading behaviors in various nanovesicles. Using our model system of PMOXA-*b*-PDMS-*b*-PMOXA polymersomes loaded with model cargoes of increasing hydrophilicity, we could distinguish core and membrane loading behavior by analyzing the relationship between carrier and cargo peaks across the particle population. We could also detect different cargo loading subpopulations by applying NNMF clustering followed by RMSE evaluation of the difference between the two clusters. However, the exact parameters, such as the gradient to distinguish between core and membrane loading, will likely depend on the Raman scattering and loading amount for specific nanocarrier/cargo systems. We found that our model cargoes behaved similarly in liposomal nanocarriers, which validated that our model could be applied to different nanoformulation systems. We also showed that our analytical framework is applicable to clinically relevant systems, by comparing the different release of chloroquine from DPPC:CH liposomes and doxorubicin from the FDA-approved formulation Doxil. We envision that our method can be widely applied to analyze nanoformulations, both for formulation development and quality control, for example, to compare different storage conditions, or stability of distinct nanoformulation compositions. We anticipate that this method can eventually assist manufacturers in quality control and aid regulatory bodies to contribute to the approval of more nanoformulations and translation to the clinic.

## Materials and Methods

### Polymersome Preparation

Polymersomes were prepared by thin film hydration and extrusion. For particles loaded with **Et** and **COOMe**, PMOXA-*b*-PDMS-*b*-PMOXA (poly(2-methyl-2-oxazoline)-*b*-poly(dimethylsiloxane)-*b*-poly(2-methyl-2-oxazoline) triblock copolymer *M*_n_ 10^3^ = 0.9–4.8–0.9, P11474-MOXZDMSMOXZ, Polymer Source, Quebec, Canada) and the cargo were dissolved in chloroform at 10 and 2 mg/mL respectively. For particles loaded with **NH**_**2**_, polymer and cargo were dissolved in THF at 10 mg/mL and either 1 or 2 mg/mL, respectively. Films were made by pipetting 3 mg of polymer and either 0.1, 0.5, 1, 1.5, or 2 μmol of cargo into separate 1.75 mL glass vials. Chloroform was removed by a stream of N_2_ above the surface for ∼10 min followed by vacuum desiccation for 1 h. The films were then hydrated with 1 mL of DPBS (14190144, ThermoFisher Scientific UK) with stirring for 24 h. For particles loaded with **SO**_**3**_ and **NMe**_**3**_, PMOXA-*b*-PDMS-*b*-PMOXA was dissolved at 10 mg/mL in chloroform, and 3 mg of polymer was pipetted into separate 1.75 mL glass vials. Chloroform was again removed under a stream of N_2_ above the surface for ∼10 min, followed by vacuum desiccation for 1 h. Films were rehydrated with 1 mL of cargo solutions at 0.1, 0.5, 1, 1.5, or 2 mM. Polymersomes suspensions were then extruded sequentially 21 times through polycarbonate membranes with mesh size 400 nm, 200 and 100 nm (Whatman Nucleopore Track-Etched Membrane). Polymersomes loaded with all cargoes were purified from free cargo by size exclusion chromatography using a PD MidiTrap column (GE Healthcare) equilibrated DPBS.

### DLS and ζ Potential

Measurements (*n* = 3 technical repeats) were made with a Malvern Zetasizer Nano-ZS. For DLS, 10 μL of particle suspension was diluted with 90 μL of DPBS in single use microcuvettes (Brand GMBH, Germany) and measured by NIBS at 173° scattering angle. The ζ potential measurements were performed on 100 μL of particle suspension in 900 μL of 0.3 M sucrose. DLS intensity curves and ζ potentials were the average of 3 technical repeats.

### Cryo-TEM

Four μL sample aliquots (4 μL, 1–2 mg/mL) were adsorbed onto a holey carbon-coated grid (Lacey, Tedpella, USA), blotted with Whatman 1 filter paper, and vitrified into liquid ethane at −180 °C using a Leica GP2 plunger (Leica microsystems, Austria). The frozen grids were then transferred onto a Talos L120C Electron microscope (FEI, USA) using a Gatan multispecimen cryo-holder Model 910 (Gatan, USA). An accelerating voltage of 120 kV using a low-dose system (40 e^–^/Å^2^) was used to record electron micrographs, while the sample was kept at −175 °C. Defocus values were −2 to 3 μm. A 4K × 4K Ceta CMOS camera was used to record electron micrographs.

### Neutron Scattering

Empty polymersomes and polymersomes loaded with 2 mM **Et** and **COOMe** were prepared, as described previously. Samples then underwent buffer exchange to deuterated PBS for SANS measurements (Gibco PBS tablets (ThermoFisher Scientific) dissolved in D_2_O). Samples in PBS were passed through a PD MidiTrap column (GE Healthcare) equilibrated in deuterated PBS. All of the measurements were performed at the ZOOM beamline of the ISIS Pulsed Neutron Source at the Rutherford Appleton Laboratory, Didcot, UK. A sample changer and 2 mm path length quartz cuvette cells were used. The beamline was configured with L1 = L2 = 4 m, where L1 is the source to sample distance and L2 is the sample to detector distance, yielding a scattering variable (*Q*) range of 0.004 to 1 Å^–1^. Samples were measured at 15 μAmps (SANS) and 5 μAmps (TRANS) at 25 °C.

SANS data were reduced with MantidPlot.^53^ SasView v5.0.4. (http://www.sasview.org/) was employed to fit the experimental data over a *q* range of 0.01 < *q* < 0.1 Å^–1^ with a polydispersity of 0.1 on the radius and shell thickness. Scattering length density (sld) of core, shell, and solvent were set to to 6.3, 4, and 6.3 × 10^–6^/Å^2^, respectively.

### SPARTA Measurements and Spectral Analysis

SPARTA measurements were performed either on a custom system as previously described or on the SPARTA alpha prototype.^29,44^ 200 μL of particle suspension was placed on a 22 mm diameter coverglass (VWR) affixed to a microscope slide, and a 63× 1.0 NA water immersion lens (W Plan Apochromat, Zeiss) was immersed in the droplet. For polymersomes, each trapped particle was measured for 20 s before 1 s laser shuttering to allow particle release and diffusion of a new particle into the confocal volume. Liposomes were measured for 10 s to prevent sample aggregation under the laser beam. Twenty blank DPBS spectra were measured at 10 or 20 s, and the average of these spectra was used for background subtraction, as applicable. For the stability study, 20 blank measurements of 10% FBS were measured as background. Powders of **Et** and **COOMe** were measured by placing on a fluoride slide and using custom Matlab scripts to move the stage until the powder was focused. Then 1 acquisition at 5 s was used to obtain the powder spectra.

Raman spectra were preprocessed using custom Matlab scripts. First a spectral response correction was added, which was calculated from a relative intensity correction sample for 785 nm excitation from NIST (National Institute of Standards and Technology, US, SRM2241). Aggregates or empty traps were then manually removed by minimum and maximum thresholding. A background subtraction of 95% was then performed using blank DPBS, followed by Whittaker baseline correction. Each spectrum was then smoothed using a Savitzky-Golay smoothing filter with order 1 and frame size 7. Each particle spectrum was normalized to the mean intensity of the 708 cm^–1^ peak for that particular feed amount, to remove effects such as laser power fluctuation between samples.

Mean spectra and standard deviation were calculated across one feed amount in Matlab R2020a and plotted in OriginPro 2020b. Linear analysis and NNMF with 2 factors were performed using custom scripts in Matlab R2020a.

### Giant Vesicle Preparation

Giant vesicles were formed using the spontaneous formation technique.^54^ 7 mL glass vials were plasma treated for 1 min. For control (without loaded compound) and **SO**_**3**_-loaded giant polymersomes, a mixture of 50 μL 20 mg/mL PMOXA-*b*-PDMS-*b*-PMOXA (500-4800-500 Da, P18140D-MOXZDMSMOXZ, Polymer Source, Quebec, Canada) with 8 μL 20 mg/mL PDMS-heparin, both in ethanol, was used.^54^ For **NH**_**2**_-loaded giant polymersomes, 50 μL of 20 mg/mL copolymer PMOXA-*b*-PDMS-*b*-PMOXA (900-4800-900 Da, P11474-MOXZDMSMOXZ, Polymer Source, Quebec) and 14 μL of 43 mM **NH**_**2**_ compound were mixed in the vial, both in THF. The solvent was left evaporating at room temperature. To further dry the films, they were put in a desiccator for at least 1 h. The polymer films were then hydrated with 0.6 mL of 0.3 M sucrose containing either nothing (control and **NH**_**2**_-giants, compound was already in the film) or 0.6 mL with 5 mM **SO**_**3**_-compound dissolved in 0.3 M sucrose and pH adjusted to 7 with NaOH. The hydrated films were put at 60 °C under static condition overnight. Then, using a 1 mL pipet tip, the giant vesicles were forcefully pipetted away from the glass surface and then transferred and immobilized as described below.

### Raman Imaging of Giant Polymersomes

To immobilize giant vesicles, calcium fluoride slides were pretreated with 10 mg/mL protamine (Protamine sulfate salt from salmon, Sigma) for 10 min. After 5 washes with DPBS, 20 μL of vesicle suspension was incubated on slides for 1 h, before 20 washes with DPBS. Vesicles were imaged using the alpha 300R+ confocal Raman microscope (Witec GmBH, Germany). A 35 mW, 532 nm laser light source was shone through a 63× 1.0 NA water immersion objective lens (W Plan-Apochromat, Zeiss, Germany). Raman scattering was collected through the same lens and directed via a 100 μm diameter silica fiber to a 600 groove/mm spectrograph (UHTS 300, WITec, GmbH, Germany) coupled to a back-illuminated charge-coupled device camera, cooled to −60 °C. Area scans of vesicles were imaged with 500 × 500 XY nm resolution. Spectral preprocessing was performed with ProjectFIVE software (Witec GmBH). First, cosmic rays were removed, and then the dark current background was subtracted, followed by “shape” background correction. Spectra were normalized to the maximum intensity of the water peak at 3000–3400 cm^–1^. Finally, Raman images were reconstructed from univariate analysis of the intensity of the **NH**_**2**_ signal at 2208 cm^–1^ and the **SO**_**3**_ signal at 2220 cm^–1^. Due to low intensity of the 708 cm^–1^ polymer peak with this instrumental setup, Raman images of polymer signal were reconstructed from 2905 cm^–1^ polymer peak.

### Relative Raman Intensity of Model Cargoes Compared to EdU

Stocks of 4 mM of each model cargo in DMSO were mixed with either 100 mM EdU in DMSO and DMSO 0.8:1:0.2 v:v:v or with 80 mM EdU 1:1 v:v. On an alpha 300R+ confocal Raman microscope (Witec GmBH, Germany) with a 532 nm laser, 100 μL of each cargo:EdU mixture was measured at 1 s for 10 acquisitions. In the ProjectFIVE software, spectra were preprocessed by cosmic ray removal, spectral cropping to 1903–2500 cm^–1^, dark current subtraction, “shape” background correction and normalization to the EdU peak intensity at 2108 cm^–1^.

### Thin-Film Preparation and Raman Imaging

Into a glass vial, 300 μL of PMOXA-*b*-PDMS-*b*-PMOXA (poly(2-methyl-2-oxazoline)-*b*-poly(dimethylsiloxane)-*b*-poly(2-methyl-2-oxazoline) triblock copolymer *M*_n_ 10^3^ = 0.9-4.8-0.9, P11474-MOXZDMSMOXZ, Polymer Source, Quebec, Canada) at 10 mg/mL in chloroform, and either 129 or 159 μL of **Et** or **COOMe** at 1 mg/mL in chloroform, or 116 μL of **NH**_**2**_ at 1 mg/mL in THF was pipetted. Solvent was evaporated off for 15 min under a stream of nitrogen. Polymer/**Et** and polymer/**COOMe** samples were then solvated in 100 μL of chloroform, and the polymer/**NH**_**2**_ sample was solvated in 100 μL of 1:1 (v:v) THF:chloroform. Onto separate calcium fluoride slides each sample was then added 20 μL at a time and dried for 3 h at room temperature. Samples were then dried under vacuum for a further 2 h until measurement.

Films were imaged on setup described above with an acquisition time of 0.2 s over a 30 × 30 μm area with 0.5 μm XY resolution. Spectra were then preprocessed using ProjectFIVE software (Witec GmBH) to remove cosmic rays, subtract dark current, perform “shape” baseline correction, and normalize to the area under the curve. Raman images were reconstructed from univariate analysis of the 2905 cm^–1^ peak area (polymer), 2213 cm^–1^ (**Et** and **COOMe** dialkyne) and 2204 cm^–1^ (**NH**_**2**_ dialkyne) peak areas.

### Liposome Preparation

Films of 4:1 mol % DPPC:CH were prepared by adding 265 μL of a 10 mg/mL DPPC (Avanti, 850355P-200MG) stock in chloroform and 34 μL of a 10 mg/mL CH stock in chloroform (Sigma) to a glass vial. For **Et** and **NH**_**2**_ loaded particles, 1 μmol of **Et** or **NH**_**2**_ in either chloroform or THF, respectively, at 1 mg/mL was added to glass vial. Chloroform was removed by a stream of nitrogen above the solution surface for 10 min, followed by 1 h of vacuum desiccation. **SO**_**3**_ and chloroquine loaded liposomes were rehydrated with either 1 mL of 1 mM **SO**_**3**_ in DPBS (no Ca, no Mg, Sigma) or 50 mM chloroquine in ultrapure water. After 5–6 freeze–thaw cycles, the suspension was extruded through a 100 nm pore-size membrane using an Avanti mini-extruder. Liposomes were purified by running 2× SEC columns through a PD MidiTrap column (GE Healthcare) equilibrated DPBS.

### Stability Studies

CQ-lip were diluted 2x with DPBS into a Spectra-Por Float-a-Lyzer G2 (Spectrum Laboratories) with 1000 kDa MWCO to a final volume of 1 mL. The sample was dialyzed in 300 mL DPBS with 1% penicillin-streptomycin (P/S) v:v for 48 h at 37 °C. At 2, 4, 9, 24, and 48 h, samples were taken from within the dialysis tube for DLS and SPARTA characterization. DLS measurements were taken by diluting 10× in DPBS. For SPARTA measurements, samples were diluted 10–30× in DPBS and measured with an acquisition time 10 s per particle. Samples were preprocessed as described above with a blank DPBS background subtraction. Doxil was diluted 5× with an experimental buffer containing 10% fetal bovine serum (FBS) and 1% P/S in DPBS (v:v) to a total volume of 1 mL in a Spectra-Por Float-a-Lyzer with 300 kDa MWCO. The sample was then dialyzed in the same 10% FBS + 1% P/S buffer at 37 °C for 48 h, with samples taken at 2, 4, 9, 24, and 48 h for DLS and SPARTA characterization. DLS measurements were taken by diluting 10× in DPBS because FBS has a strong DLS background. SPARTA measurements were made by diluting samples in the same 10% FBS experimental buffer with a 10 s acquisition time. SPARTA measurements were preprocessed in the same manner as previously described using a blank background of the 10% FBS experimental buffer.

## Supporting Information Available

Synthesis of model cargo compounds. SANS fitting parameters. Supporting Figures 1–8: cryo-TEM overview images; repeats of **NH_2_**, **SO_3_** and **NMe_3_** loading into PMOXA-*b*-PDMS-*b*-PMOXA; additional images from Raman confocal spectroscopy of giant polymersomes loaded with model cargoes; scatter plots of model cargoes loaded into polymersomes prepared from different copolymers; confocal Raman imaging of films prepared from PMOXA-*b*-PDMS-*b*-PMOXA and 0.5 mM **Et**, **COOMe** or **NH_2_**; DLS of liposomes loaded with model cargoes. Theoretical modeling of cargo location (PDF)
