# Supplementary material for: Revealing Population Heterogeneity in Vesicle-Based Nanomedicines Using Automated, Single Particle Raman Analysis
Source: ACS Nano. 2023 Jun 6;17(12):11713–28. doi: 10.1021/acsnano.3c02452 (PMC10311594; doi:10.1021/acsnano.3c02452)
Supplement: Supplementary file 1 — nn3c02452_si_001.pdf [file nn3c02452_si_001.pdf]

<u>Supporting Information</u>

Revealing population heterogeneity in vesicle-based nanomedicines using automated, single particle Raman analysis

Catherine Saunders[1], James E. J. Foote[1], Jonathan P. Wojciechowski[1], Ana Cammack[1], Simon V. Pedersen[1], James J. Doutch[2], Hanna M. G. Barriga[3], Margaret N. Holme[3], Jelle Penders[1], Mohamed Chami[4], Adrian Najer[1]*, Molly M. Stevens[1,3]*

[1] Department of Materials, Department of Bioengineering, and Institute of Biomedical Engineering, Imperial College London, London SW7 2AZ, U.K.

[2] ISIS Neutron and Muon Source, Rutherford Appleton Laboratory, STFC, Didcot OX11 ODE, U.K.

[3] Department of Medical Biochemistry and Biophysics, Karolinska Institutet, SE-171 77 Stockholm, Sweden

[4] BioEM lab, Biozentrum, University of Basel, Mattenstrasse 26, 4058, Basel, Switzerland

*Correspondence to a.najer@imperial.ac.uk and m.stevens@imperial.ac.uk

Model Cargo Synthesis and Characterisation

Chemicals were purchased from Sigma-Aldrich, Fluorochem and VWR. Reagents were used as received without further purification.

Unless otherwise stated, ^{1}H and ^{13}C NMR spectra were measured on a JEOL 400 MHz NMR spectrometer or a Bruker Avance III HD 600 MHz NMR spectrometer equipped with a triple-resonance cryoprobe. NMR spectra were recorded at 298 K, with the samples dissolved in the solvents stated, and the chemical shifts referenced to the residual solvent resonance (δ = 7.26 ppm, $CDCl_3$) or to an internal standard (δ = 0 ppm, 3-(trimethylsilyl)propionic-2,2,3,3-d_4 acid sodium salt) for samples dissolved in D_2O.

LC-MS chromatograms and ESI-MS spectra were measured on an Agilent 1260 Infinity Quaternary LC with an Agilent 6130 Quadrupole ESI-MS. Samples were measured using a Phenomenex Kinetex Evo C18 column (50 $\times$ 2.1 mm, 2.6 µm, 100 Å) at a flow rate = 0.5 mL/min, injection volume = 5 µL, detection wavelengths = 220 nm and 254 nm, and column temperature = 313 K. Mobile phases were: A = HPLC grade water with 0.1% formic acid (v/v) and B = HPLC grade acetonitrile with 0.1% formic acid (v/v). Samples were dissolved in mixtures of acetonitrile/water with 0.1% formic acid (v/v). The samples were separated using a gradient elution from 5-95% B over 12 min.

Preparative RP-HPLC was performed using a Shimadzu Prominence 20A with a Phenomenex Gemini NX C18 column (151 $\times$ 21.2 mm, 5 mm, 110 Å) at a flow rate = 10 mL/min, injection loop = 20 mL and, detection wavelengths = 220 and 280 nm. Mobile phases were: A = HPLC grade water with 0.1% formic acid and B = HPLC grade acetonitrile with 0.1% formic acid. Samples were separated using a gradient elution from 5-95% B over 30 min.

UV-vis spectra were measured using a Thermo Scientific™ NanoDrop™ 2000c Spectrophotometer in cuvette mode with a 10 mm pathlength quartz cuvette.

Et

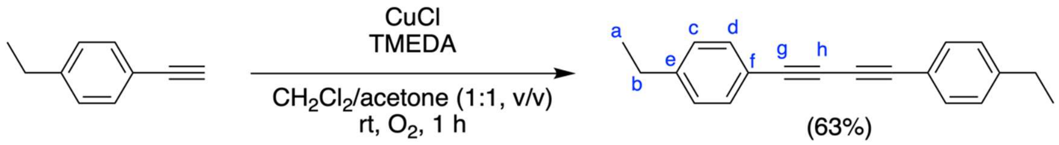

To a 14 mL glass vial was added copper (I) chloride (74.1 mg, 0.75 mmol, 1 eq) and a magnetic stirrer bar. Then a mixture of 7 mL chloroform/acetone (1:1, v/v) was added, followed by *N*,*N*,*N'*,*N'*-tetramethylethylenediamine (232 µL, 1.53 mmol, 2 eq), giving a bright green solution. The reaction mixture was bubbled with air for 5 min, yielding a dark blue solution. Then 4-ethylphenylacetylene (105 µL, 0.77 mmol, 1 eq) was added and reaction mixture was stirred for 1 h at room temperature, forming a dark grey solution. The reaction was quenched with 6 mL of hexane and filtered to remove precipitated catalyst. The filtrate was collected, dry loaded onto celite *via* rotary evaporation, and then purified using flash column chromatography using a gradient from hexane to 20 % ethyl acetate in hexane. The product was isolated as a light yellow powder (63 mg, 63%).

^{1}H NMR (400 MHz, Chloroform-*d*): δ 7.44 (t, 4H, *J* = 7.8 Hz, H^d), 7.15 (d, 4H, *J* = 8.0 Hz, H^c), 2.65 (q, 4H, *J* = 7.3 Hz, H^b), 1.23 (t, 6H, *J* = 6.8 Hz, H^a).

^{13}C NMR (100 MHz, Chloroform-*d*): δ 145.84 (C^e), 132.59 (C^d), 128.12 (C^c), 119.13 (C^f), 81.67 (C^g), 73.55 (C^h), 29.01 (C^b), 15.32 (C^a).

This agrees well with previously reported assignments for this compound.[1,2]

COOMe

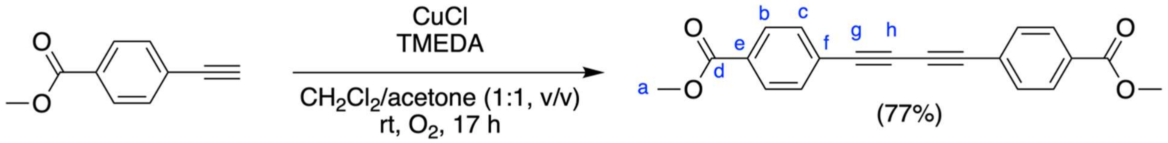

To a round bottom flask (50 mL) was added methyl 4-ethynylbenzoate (168.6 mg, 1.05 mmol, 1 eq), copper (I) chloride (10.6 mg, 0.107 mmol, 0.1 eq), and a magnetic stirrer bar. Then, 10 mL of dichloromethane/acetone (1:1, v/v), followed by *N*,*N*,*N'*,*N'*-tetramethylethylenediamine (45 µL, 0.301 mmol, 0.3 eq) was added, which gave a green colour to the reaction mixture. The reaction mixture was bubbled with air for 15 min, then allowed to react for 17 h at room temperature open to the atmosphere. There was a large amount of yellow precipitate in the reaction flask following overnight reaction. The precipitate was dissolved in dichloromethane (15 mL) and dry loaded onto celite *via* rotary evaporation. The reaction mixture was dry loaded onto a silica column and eluted using a gradient from hexane to 20% ethyl acetate in hexane. The title compound was isolated as an off-white solid (130.3 mg, 77%).

^{1}H NMR (400 MHz, Chloroform-*d*): δ 8.01 (d, 4H, *J* = 8.6 Hz, H^b), 7.59 (d, 4H, *J* = 8.6 Hz, H^c), 3.93 (s, 6H, H^a).

^{13}C NMR (100 MHz, Chloroform-*d*): δ 166.40 (C^d), 132.62 (C^e), 130.70 (C^c), 129.73 (C^b), 126.26 (C^f), 81.99 (C^g), 76.40 (C^h), 52.49 (C^a).

MS (ESI-MS, *m/z*): calculated for $C_{20}H_{14}O_4$ $[M+H]^+$: *m/z* = 319.1; found 319.1.

The data agrees well with previously reported assignments for the compound.[3]

NH_2

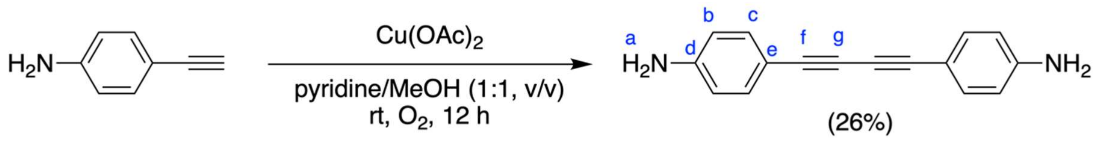

To a reaction vessel was added 4-ethynylaniline (200 mg, 1.71 mmol, 1 eq), a magnetic stirrer bar and 20 mL of pyridine/methanol (1:1, v/v), followed by copper (II) acetate (775.2 mg, 4.27 mmol, 2.5 eq). The reaction mixture was bubbled with air then stirred for 12 h at room temperature. The reaction was purified by recrystallisation in 3 mL acetone/water (1:2, v/v) to yield the product as a white/yellow powder (52.3 mg, 26%).

^{1}H NMR (400 MHz, DMSO-*d6*): δ 7.19 (d, 4H, J = 7.2 Hz, H^c), 6.52 (d, 4H, J = 6.5 Hz, H^b), 5.70 (br s, 4H, H^a).

^{13}C NMR (100 MHz, DMSO-*d6*): δ 150.15 (C^d), 133.50 (C^c), 113.50 (C^b), 106.40 (C^e), 82.88 (C^f), 71.99 (C^g).

MS (ESI-MS, *m/z*): calculated for $C_{16}H_{12}N_2$ $[M+H]^+$: *m/z* = 233.1; found 233.1.

The data agrees well with previously reported assignments for the compound.[1,4]

NMe_3

NMe_2

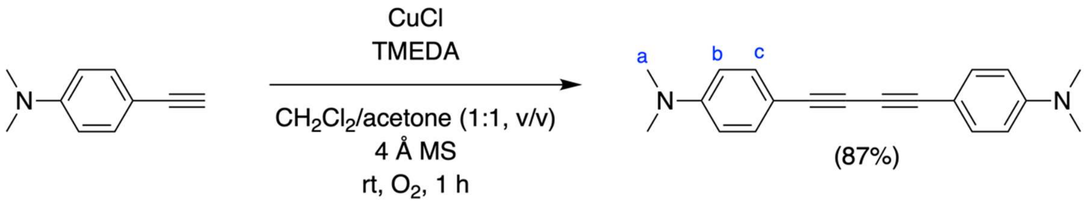

Compound **NMe_2** was synthesised following literature procedures.[5]

To a 7 mL glass vial was added a magnetic stirrer bar, copper (I) chloride (9.9 mg, 0.100 mmol, 0.1 eq), *N*,*N*,*N′*,*N′*-tetramethylethylenediamine (45 µL, 0.301 mmol, 0.3 eq), activated 4 Å molecular sieves (1.5 g) and 4 mL of dichloromethane/acetone (1:1, v/v). The reaction mixture was stirred vigorously open to air for 10 min, then 4-ethynyl-*N*,*N*-dimethylaniline (148 µL, 0.999 mmol, 1 eq) was added. The reaction was stirred at room temperature for 1 h. The reaction mixture was then transferred to a round bottom flask with dichloromethane (50 mL) and dry loaded onto silica. The crude reaction mixture was purified using flash column chromatography using a gradient starting from hexane to 20% ethyl acetate in hexane. The title compound was isolated as a light yellow-brown solid (125.8 mg, 87%).

^{1}H NMR (400 MHz, Chloroform-*d*): δ 7.40 (d, 4H, J = 9.0 Hz, H^c), 6.66 (br d, 4H, J = 8.3 Hz, H^b), 3.00 (s, 12H, H^a).

MS (ESI-MS, *m/z*): calculated for $C_{20}H_{20}N_2$ $[M+H]^+$: *m/z* = 289.2; found 289.1.

The data agrees well with previously reported assignments for the compound.[6]

NMe_3

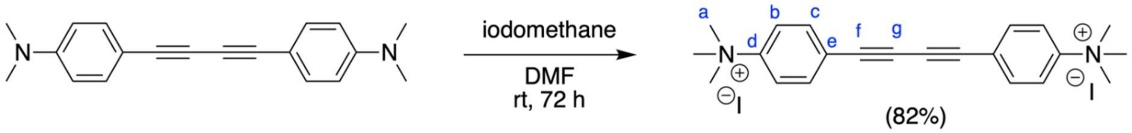

To a 7 mL glass vial was added a magnetic stirrer bar, **NMe_2** (50.3 mg, 0.174 mmol, 1 eq) and *N*,*N*-dimethylformamide (3 mL). Then, iodomethane (0.542 mL, 8.72 mmol, 50 eq) was added to the reaction mixture, which was stirred at room temperature for 72 h. The reaction mixture was quenched with methanol (2 mL), stirred for 30 min, then precipitated into diethyl ether (30 mL) and centrifuged (5000 rcf, 5 min) to collect the precipitate. The precipitate was washed with diethyl ether (3x 30 mL) *via* suspension and centrifugation, then the precipitate was dried with a gentle stream of nitrogen. The precipitate was dissolved in 5% acetonitrile in water with 0.1% formic acid and purified using Prep-HPLC using a 5-95% B gradient over 30 min. The fractions containing the desired product were lyophilised to give the title compound as a yellow solid (81.9 mg, 82%).

^{1}H NMR (600 MHz, D_2O): δ 7.89 (d, 4H, *J* = 9.4 Hz, H^b), 7.84 (d, 4H, *J* = 9.4 Hz, H^c), 3.68 (s, 18H, H^a).

^{13}C NMR (151 MHz, D_2O): δ 149.70 (C^d), 137.21 (C^c), 126.28 (C^e), 123.16 (C^b), 83.07 (C^f), 77.94 (C^g), 59.77 (C^a).

UV-Vis (H_2O): λ_{max}/nm 290 ($\varepsilon = 8.5 \times 10^3$ M^{-1} cm^{-1}), 308 ($\varepsilon = 1.2 \times 10^4$ M^{-1} cm^{-1}), 330 ($\varepsilon = 1.0 \times 10^{-4}$ M^{-1} cm^{-1}).

HR-MS (ESI-MS, *m/z*): calculated for $C_{22}H_{26}N_2$ $[M]^{2+}$: *m/z* = 159.1043; found 159.1043.

<u>SO3</u>

2,2,2-trifluoroethyl 4-iodobenzenesulfonate

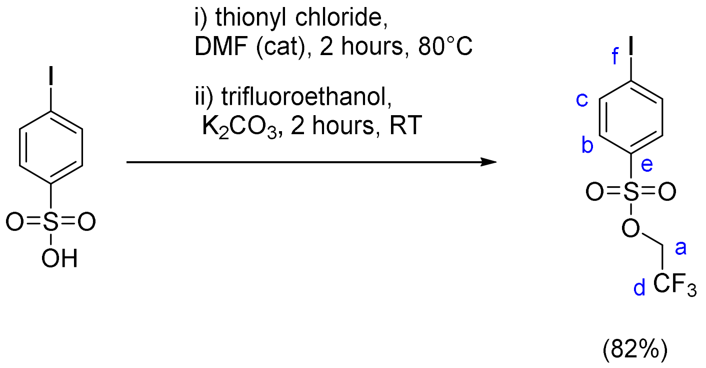

In a round bottom flask, 4-iodobenzenesulfonic acid (1.07 g, 3.78 mmol, 1 eq) was dissolved in thionyl chloride (3.37 g, 28.3 mmol, 7.5 eq), placed in an ice bath, then *N*,*N*-dimethylformamide (0.36 mL) was added. The reaction was allowed to warm to room temperature then was heated at 80 °C for 2 h. The reaction was fully dried *in vacuo* before the addition of 2,2,2-trifluoroethanol (2.88 mL, 38.1 mmol, 10 eq) and potassium carbonate (1.306 g, 9.45 mmol, 2.5 eq). The reaction was allowed to stir for 2 h at room temperature. Then, the reaction was dried *in vacuo*, dissolved in dichloromethane (100 mL) and washed with water (2x40 mL) and with brine (1x40 mL). The resultant organic layer was dried with anhydrous magnesium sulphate, filtered, and dried *in vacuo* to give the title compound white solid (1.14 g, 82%).

^{1}H NMR (600 MHz, $CDCl_3$): δ 7.96 (d, 2H, J = 8.6 Hz, H^b), 7.63 (d, 2H, J = 8.6 Hz, H^c), 4.39 (q, 2H, J = 7.9 Hz, H^a).

^{13}C NMR (150 MHz, $CDCl_3$): δ 139.03 (C^c), 134.80 (C^e), 129.32 (C^b), 121.89 (q, J =278.0 Hz, C^d), 102.88 (C^f), 64.86 (q, J = 38.3 Hz, C^a).

^{19}F NMR (400 MHz, $CDCl_3$): δ –73.69 (F^a).

MS (ESI-MS, *m/z*): calculated for $C_8H_6F_3IO_3S$ [M+HCOOH-H]$^-$: *m/z* = 410.9; found 410.8. calculated for $C_8H_6F_3IO_3S$ [M+TFA-H]$^-$: *m/z* = 478.9; found 478.8.

2,2,2-trifluoroethyl 4-((triisopropylsilyl)ethynyl)benzenesulfonate

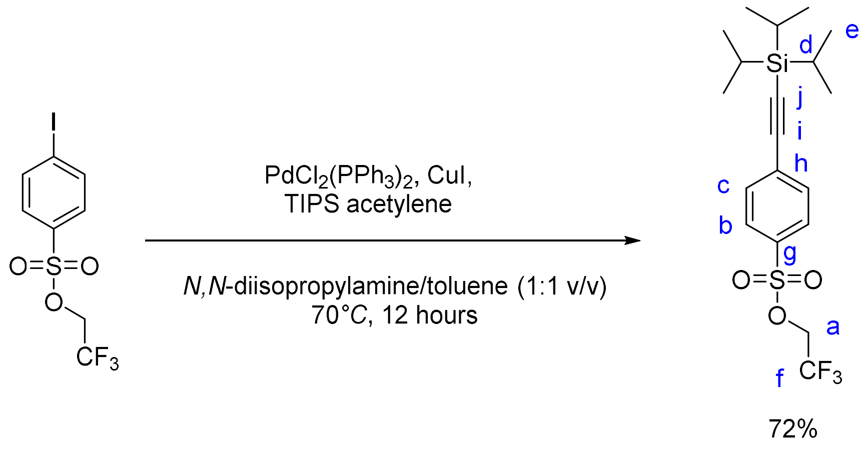

To a vial was added 2,2,2-trifluoroethyl 4-iodobenzenesulfonate (1.14 g, 3.11 mmol, 1.0 eq), bis(triphenylphosphine)palladium(II) dichloride (0.209 g, 0.156 mmol, 0.05 eq), copper(I) iodide (0.118 g, 0.622 mmol, 0.2 eq) and a magnetic stirrer bar. The vial was sealed with a septum, then anhydrous degassed toluene (4 mL) and degassed *N,N*-diisopropylamine (4 mL) were added. The reaction mixture was bubbled with argon for 10 min then argon purged (triisopropylsilyl)acetylene (1.75 mL, 11.8 mmol, 2.5 eq) was added, then the reaction was heated at 70 °C for 12 h. The reaction was cooled to room temperature, diluted in hexane (50 mL) and filtered through celite. The filtrate was then dried *in vacuo* and the crude reaction mixture purified using flash column chromatography using a gradient from hexane with 0.1% triethylamine to 10% ethyl acetate in hexane with 0.1% triethylamine. The reaction mixture was then dissolved in toluene (30 mL), then diethyl ether (150 mL) was added. This mixture was filtered and the filtrate collected and dried in vacuo to yield the title compound as a pale yellow oil (72%, 0.94 g).

^{1}H NMR (600 MHz, CDCl$_3$): δ 7.86 (d, 2H, J = 8.1 Hz, H^b), 7.65 (d, 2H, J = 8.3 Hz, H^c), 4.37 (q, 2H, J = 7.8 Hz, H^a), 1.14 (d, 21H, J = 3.2 Hz, H^d & H^e).

^{13}C NMR (150 MHz, CDCl$_3$): δ 134.04 (C^g), 132.96 (C^c), 130.43 (C^h), 128.04 (C^b), 121.92 (q, J =278.0 Hz, C^f) 104.72 (C^i), 97.35 (C^j), 64.83 (q, J = 38.3 Hz, C^a), 18.73 (C^e), 11.35 (C^d).

^{19}F NMR (400 MHz, CDCl$_3$): δ −73.73 (F^a).

MS (ESI-MS, *m/z*): calculated for $C_{19}H_{27}F_3O_3SSi$ $[M+H]^+$: *m/z* = 421.1; found 421.2.

2,2,2-trifluoroethyl 4-ethynylbenzenesulfonate

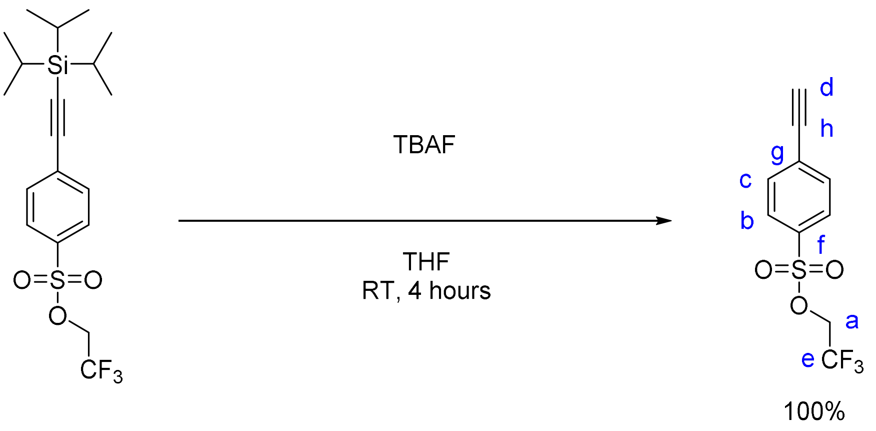

To a vial was added 2,2,2-trifluoroethyl 4-((triisopropylsilyl)ethynyl)benzenesulfonate (100 mg, 0.59 mmol, 1.0 eq) and a stirrer bar. The reaction was purged with nitrogen, then anhydrous THF (1 mL) was added. The solvent was purged with nitrogen for 10 minutes, then 1 M TBAF in THF (0.72 mL, 0.29 mmol, 1.4 eq) was added. The reaction mixture was stirred for 2 h at room temperature. The reaction was dried *in vacuo*, then redissolved in dichloromethane (50 mL) then washed with brine (50 mL), dried with anhydrous magnesium sulphate, filtered and concentrated *via* rotary evaporation. The reaction mixture was purified by flash column chromatography using (1:1 (v/v) hexane/dichloromethane with 0.1% triethylamine) to yield the title compound as a colourless oil (55 mg, quantitative).

^{1}H NMR (600 MHz, CDCl$_3$): δ 7.90 (d, 2H, J = 8.5 Hz, H^c), 7.69 (d, 2H, J = 8.5 Hz, H^b), 4.39 (q, 2H, J = 7.98 Hz, H^a), 3.33 (s, 1H, H^d).

^{13}C NMR (150 MHz, CDCl$_3$): δ 134.91 (C^f), 133.16 (C^c), 129.12 (C^g), 128.13 (C^b), 121.19 (q, J = 278.0 Hz, C^e), 82.20 (C^d), 81.60 (C^h), 64.88 (q, J = 38.2 Hz).

^{19}F NMR (400 MHz, CDCl$_3$): δ −73.74 (F$_a$).

MS (ESI-MS, *m/z*): calculated for $C_{10}H_7F_3O_3S$ $[M+H]^+$: *m/z* = 265.0; found 265.0.

SO_3

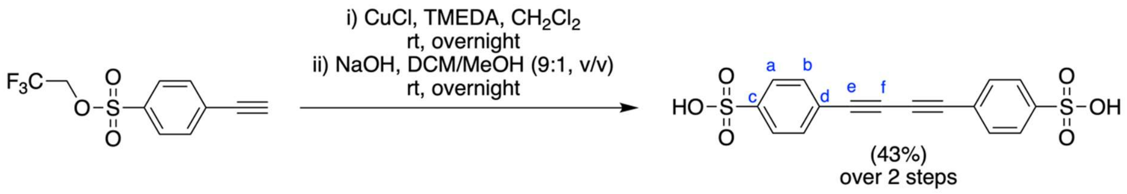

To a 7 mL glass vial was added copper (I) chloride (14.6 mg, 0.147 mmol, 0.5 eq), a magnetic stirrer bar and 2,2,2-trifluoroethyl 4-ethynylbenzenesulfonate (101.7 mg, 0.38 mmol, 1 eq). Then dichloromethane (0.5 mL) was added, followed by *N*,*N*,*N*',*N*'-tetramethylethylenediamine (85 µL, 0.567 mmol, 2 eq). After overnight stirring at room temperature in air, the crude product was dissolved in dichloromethane (3 mL) and washed with water (2x 40 mL) and brine (1x 40 mL), followed by drying over anhydrous sodium sulphate, filtration and concentration *in vacuo*. The precipitate was dry loaded onto silica and then eluted using a gradient from hexane with 0.1% triethylamine to 30% ethyl acetate in hexane with 0.1% triethylamine. The resultant solid was dissolved in dichloromethane (0.5 mL) and 2 M sodium hydroxide in methanol (50 µL) and stirred overnight at room temperature. The solution was dried under nitrogen then under vacuum and the product was purified by Preparative-HPLC to yield an off-white solid **2** (45 mg, 43%).

^{1}H NMR (600 MHz, D_2O): δ 7.84 (d, 4H, J = 7.8 Hz, H^a), 7.75 (d, 4H, J = 7.8 Hz, H^b).

^{13}C NMR (150 MHz, D_2O): δ 143.04 (C^c), 133.05 (C^b), 125.72 (C^a), 124.2 (C^d), 81.3 (C^e), 75.1 (C^f).

The data agrees well with previously reported assignments for the compound.[7]

Small Angle Neutron Scattering Fitting Parameters

Table 1. Fitting parameters from fitting SANS curves of empty, COOMe 2mM and Et 2mM loaded polymersomes with core-shell ellipsoid model using SASView v5.0.4.

	**Empty**		**COOMe**		**Et**	
	Fitted value	*Fitting Error*	*Fitted value*	*Fitting Error*	*Fitted value*	*Fitting Error*
**Scale**	0.017	7.19E-05	0.010	8.68E-05	0.017	9.91E-05
**Background (cm^{-1})**	-0.0049	0.0004	-0.0012	0.0005	-0.0032	0.0005
**radius (Å)**	837	4	833	8	846	6
**Axial ratio of core at pole to equator**	0.79	0.00	0.78	0.01	0.79	0.01
**Shell thickness (Å)**	143	1	146	1	142	1
**Ratio of shell thickness at pole to equator**	0.71	0.01	0.67	0.02	0.74	0.02

Supporting Figures

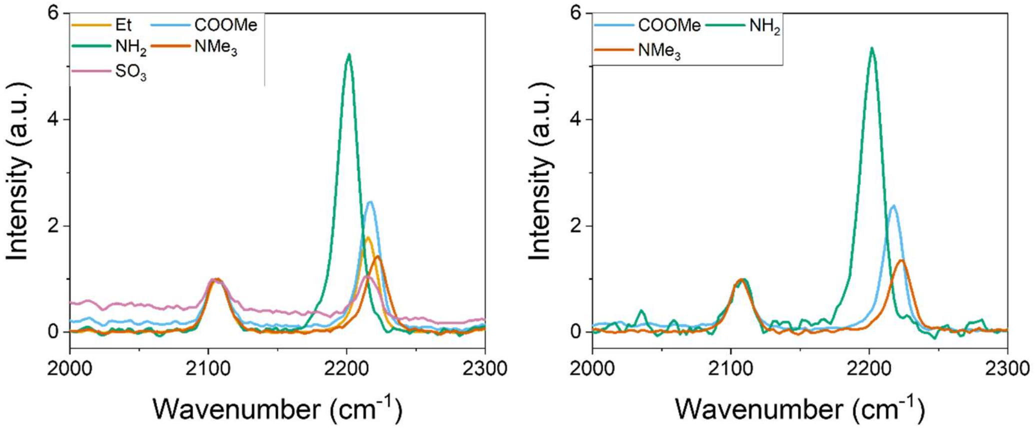

Supporting Figure S1. Additional repeats of relative Raman intensity of model cargoes. Measurements performed in DMSO with cargoes at 2 mM (peak at 2208-2225 cm^{-1}) and EdU at 40 mM. Spectra normalised to EdU peak intensity (peak at 2107 cm^{-1}). Spectra measured at 1 s for 10 acquisitions with 532 nm excitation laser.

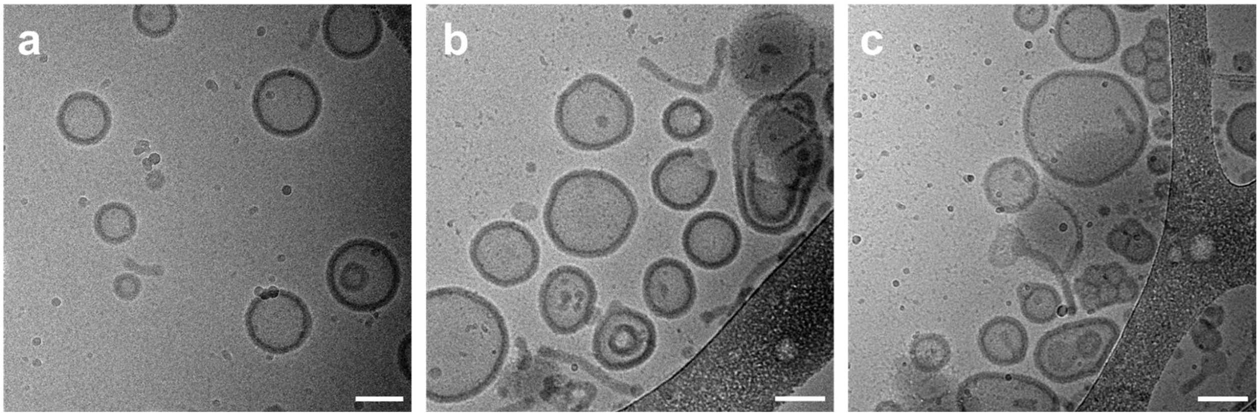

Supporting Figure S2. Polymersome morphology. Cryo-TEM images of a) empty polymersomes and polymersomes loaded with b) NH_2 and c) NMe_3. Scalebars 100 nm.

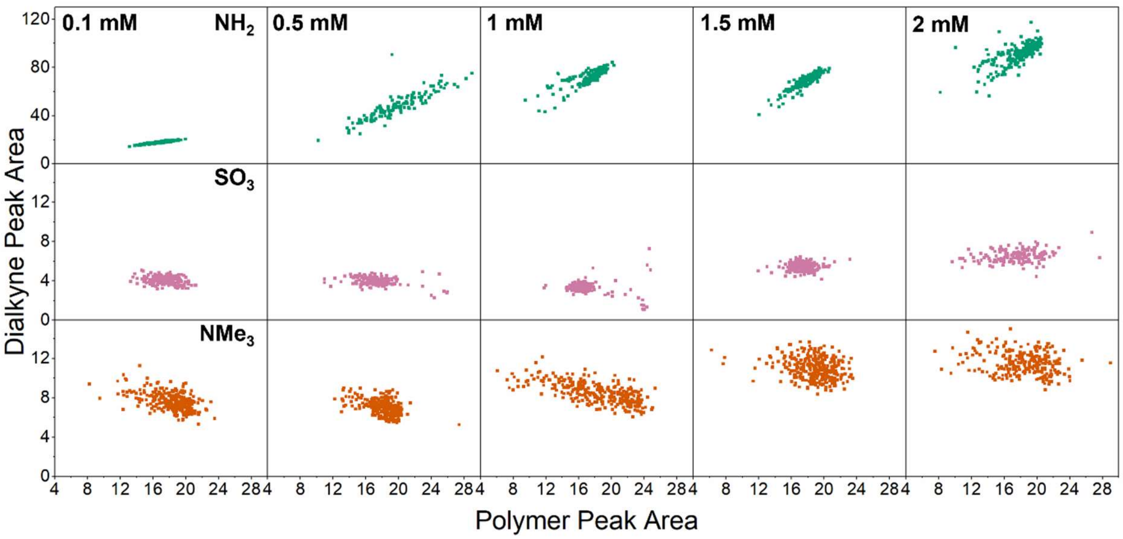

Supporting Figure S3. Polymer peak area-dialkyne peak area scatter plots. Additional repeats of loading PMOXA-*b*-PDMS-*b*-PMOXA polymersomes with NH_2 (top row, green), SO_3 (middle row, pink) and NMe_3 (bottom row, orange).

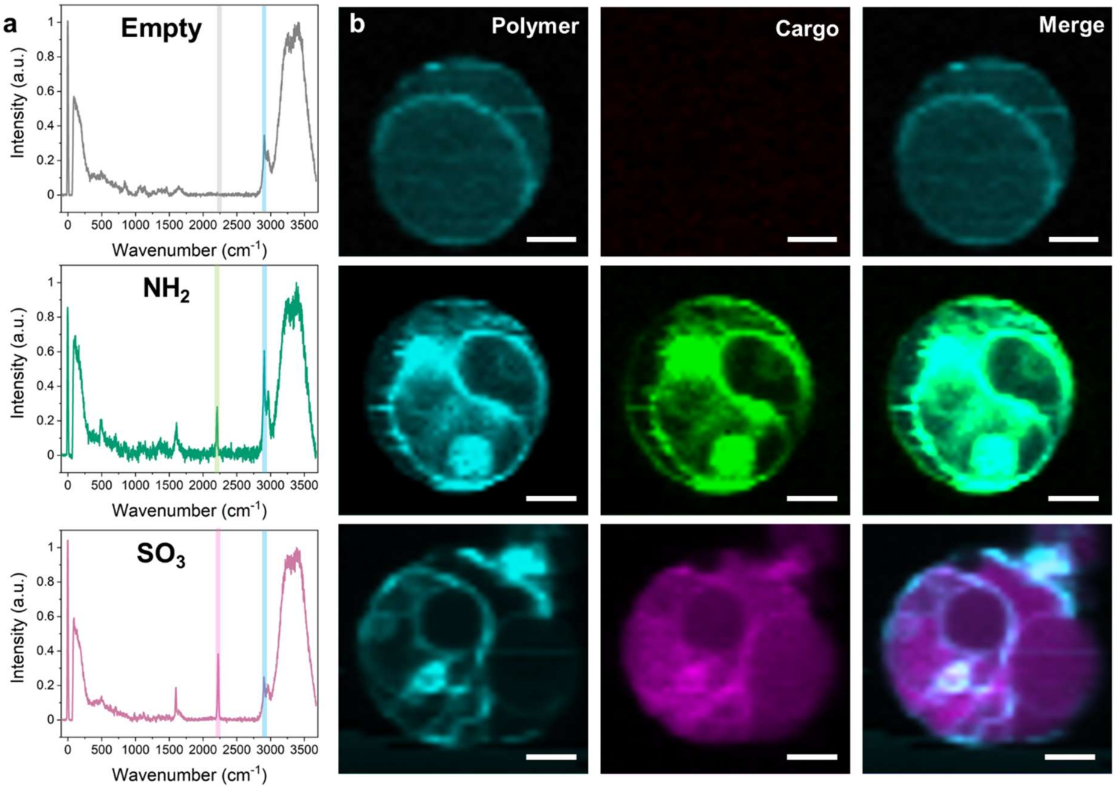

Supporting Figure S4. **Confocal Raman images of model cargo loading in additional particles**. Constructed as described in main text. a) full spectra from a representative voxel in each particle, with shaded areas indicating the peak areas used for image reconstruction. b) Raman images reconstructed from univariate analysis of polymer (2950 cm^{-1} peak area), model cargo (2208/2220 cm^{-1} peak area) and merge. Scalebars 5 µm.

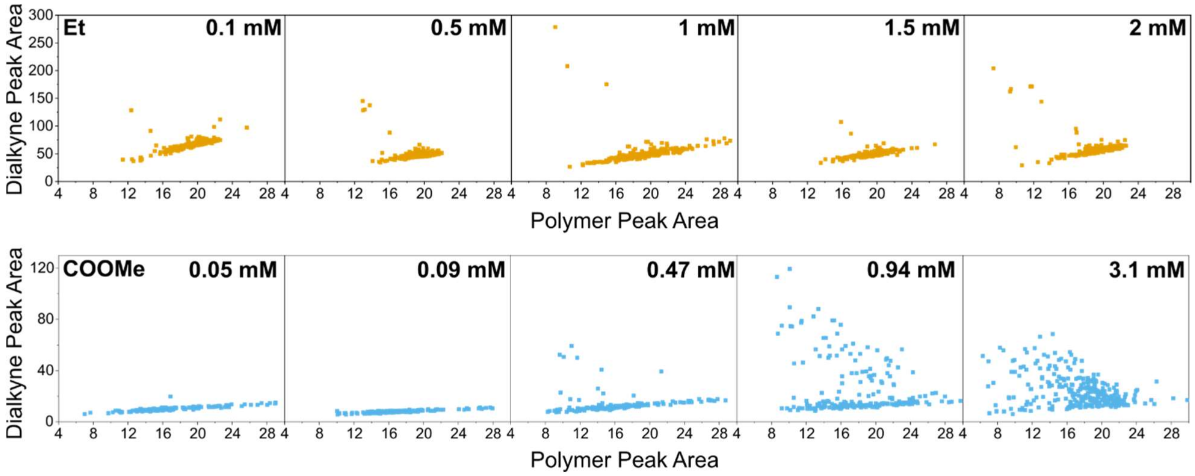

Supporting Figure S5. Scatter plots of COOMe and Et model cargo loading in additional PMOXA-*b*-PDMS-*b*-PMOXA copolymer-based polymersomes. Top row: plots of **Et** at 0.1, 0.5, 1, 1.5 and 2 mM feed amount in polymersomes prepared from a 1:1 mol:mol blend of two copolymers with 6-*b*-65-*b*-6 and 21-*b*-65-*b*-21 repeating monomer units (Polymer Source, P18140D-MOXZDMSMOXZ and P18140A-MOXZDMSMOXZ respectively). Bottom row: plots from initial loading of **COOMe** cargo at 0.05, 0.09, 0.47, 0.94 and 3.1 mM in polymersomes prepared from copolymers with 14-*b*-65-*b*-14 repeating monomer units (Polymer Source, P18140C-MOXZDMSMOXZ).

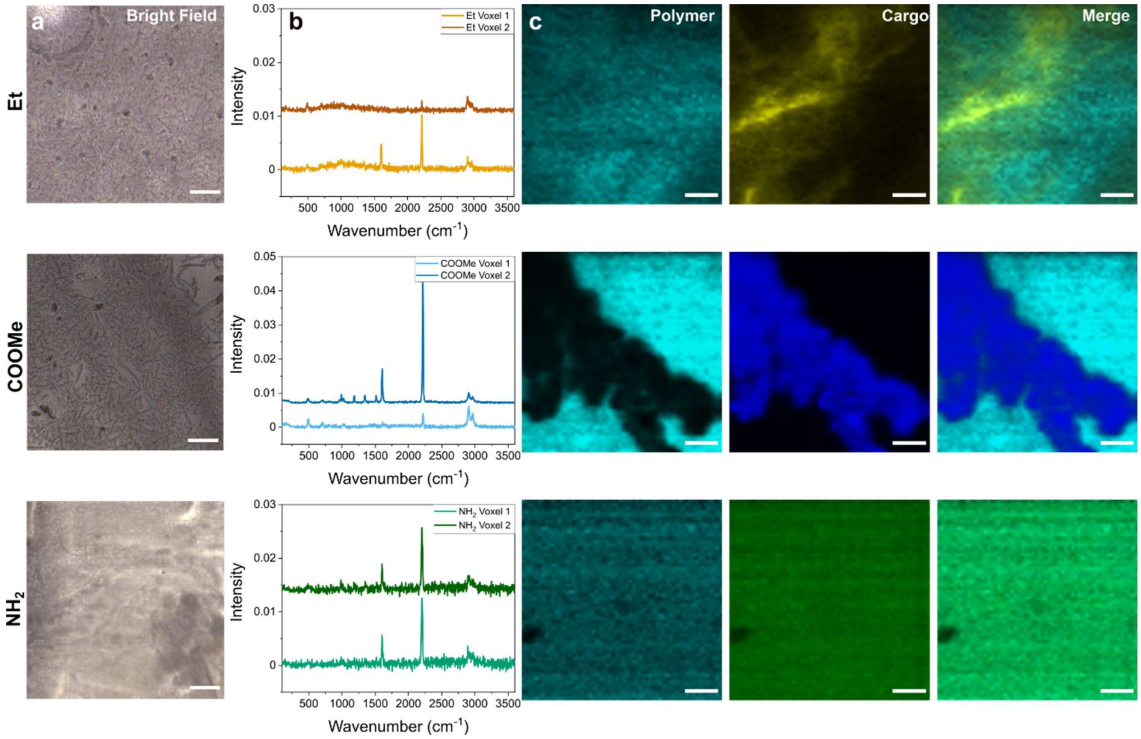

Supporting Figure S6. Confocal Raman imaging of films prepared from PMOXA-*b*-PDMS-*b*-PMOXA and 0.5 mM of either Et, COOMe or NH_2. a) Overview brightfield images of larger film area. Scalebars 100 µm. b) Representative Raman spectra from two voxels in each film. c) Raman images reconstructed from univariate analysis of 2905 cm^{-1} (polymer), 2213 cm^{-1} (Et and COOMe model cargoes) and 2204 cm^{-1} (NH_2 model cargo) peak areas. Raman image scalebars 5 µm.

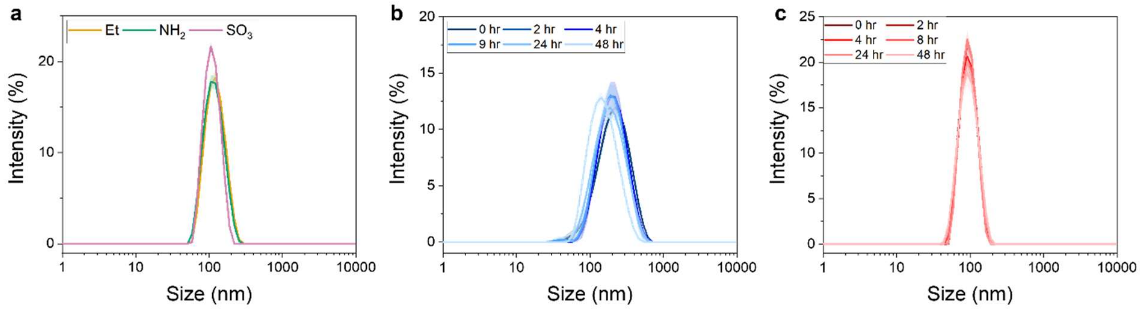

Supporting Figure S7. DLS characterisation of liposomes. DPPC:CH 4:1 mol. ratio liposomes loaded with a) model cargoes Et, COOMe and SO_3 and b) chloroquine during 48 h dialysis at 37°C. c) Characterisation of Doxil size distribution during 48 h dialysis at 37°C in 10% FBS v:v. All spectra mean ± s.d. of technical triplicates.

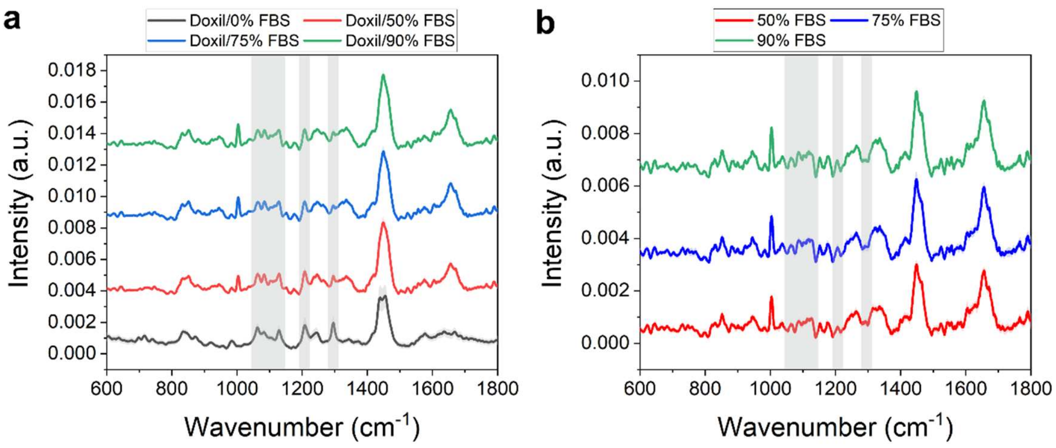

Supporting Figure S8: SPARTA measurements on Doxil in high serum conditions. Doxil was added to FBS to make a final serum concentration of 50%, 75% or 90% (v:v), and measured with SPARTA immediately. a) Mean spectra from each serum condition (n > 46, mean ± s.d.). Shaded regions show key lipid (1065-1130 cm^{-1} and 1295 cm^{-1}) and doxorubicin (1208 cm^{-1}) peaks. b) Mean solution spectra of FBS at 50%, 75% and 90% (v:v) dilutions (n > 16, mean ± s.d.). Any FBS aggregates trapped during measurement were thresholded out in preprocessing. Shaded regions from a) are repeated in b) for comparison. Spectra are normalised to the area under the curve.

Modelling Description

The aim of the modelling was to understand the variation of PDMS mass, corresponding to the 708 cm^{-1} peak, with 1 mM initial cargo mass, corresponding to the 2208/2220 cm^{-1} peak. Therefore we formed a model showing the theoretical distribution between polymer and dialkyne mass if membrane or core loading was performed.

We first found the membrane volume V_m and core volume V_c for particles of radii, r, from 25-300 nm with a 10 nm membrane thickness (Supporting Figure S9a,b)

$$V_m = \frac{4}{3}\pi[(r-10)^3] \quad (1)$$

$$V_c = \frac{4}{3}\pi[(r)^3 - (r-10)^3] \quad (2)$$

We used the number of chains per particle, N_c, to calculate polymer mass per particle. To find N_c we used the experimental value from static light scattering that there were ~10^4 PMOXA-*b*-PDMS-*b*-PMOXA chains in a 100 nm diameter polymersome,[8] which we could divide by our calculated V_m for a 100 nm diameter particle to find the expected volume per chain v_c = 2.6 x 10^{-23} L. We could then find the number of chains per particle at different radii from

$$N_c = \frac{V_m}{v_c} \quad (3).$$

We then used the known polymer molecular weight M_w and PDMS mass fraction f_{PDMS} to calculate polymer mass M_p and PDMS mass per particle M_{PDMS} (Supporting Figure S9c).

$$M_{PDMS} = f_{PDMS} \times M_P \quad (4),$$

where N_A is Avogadro's number and

$$M_P = N_c \times \frac{M_w}{N_A} \quad (5).$$

For membrane loading, the assumptions of complete, even loading into the membrane mean that membrane and cargo will be colocalised. Therefore we used the experimental weight ratio R_w for 1 mM loading to calculate the cargo mass, M_M, for a given polymer mass, where increased PDMS mass was given by unilamellar vesicles of increasing radius

$$M_M = R_w \times M_p \quad (6).$$

For core loading we again assumed cargo from the initial feed formed a homogenous solution entirely within the core volume, with no partitioning into the membrane. We therefore calculated the cargo mass per particle M_C from the experimental cargo concentration, C, and the calculated V_c (eqn 2)

$$M_C = V_c \times C \times M_r \quad (7)$$

where M_r is the cargo molar mass.

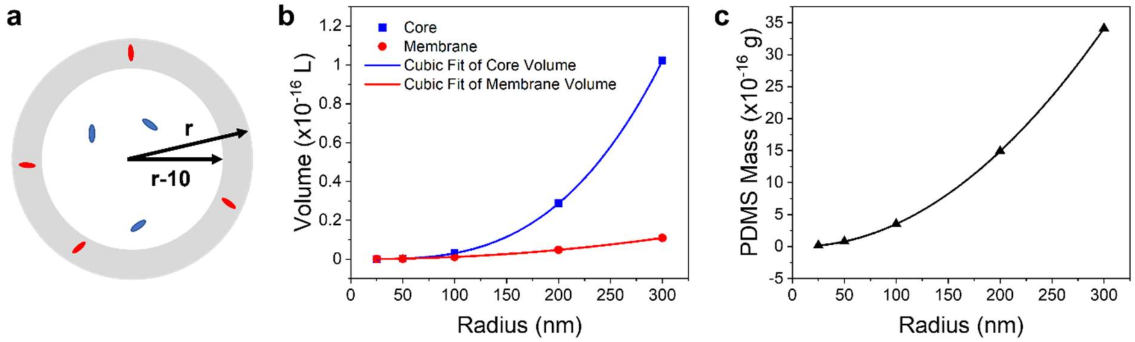

Supporting Figure S9. Theoretical modelling summary. a) Schematic showing assumed core (blue) and membrane (red) loading modes for a vesicle of radius *r*. b) Summary of change in core and membrane volumes for unilamellar vesicles with identical membrane thickness and increasing radius. c) Variation of PDMS mass with radius for simplified vesicle model. Main model predictions (variation of PDMS mass and model cargo mass) shown in main text.

NMR Appendix

2,2,2-trifluoroethyl 4-iodobenzenesulfonate

^{1}H NMR (600 MHz, $CDCl_3$)

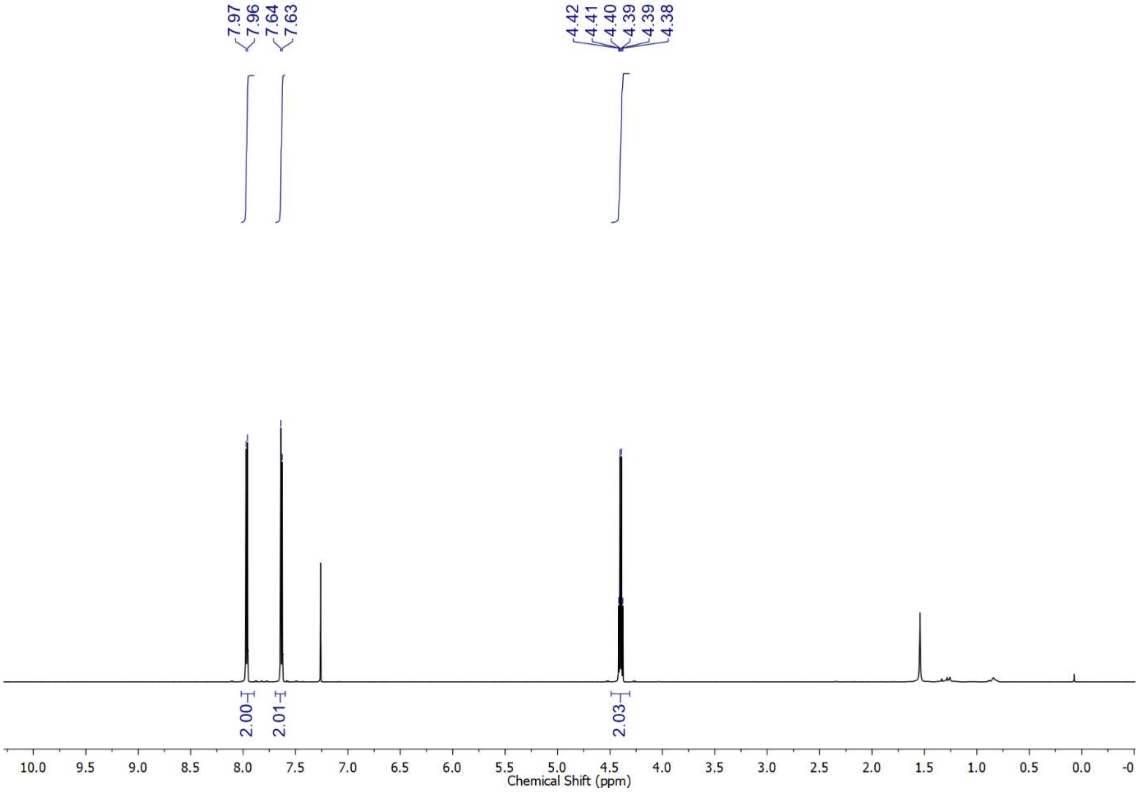

^{13}C NMR (150 MHz, $CDCl_3$)

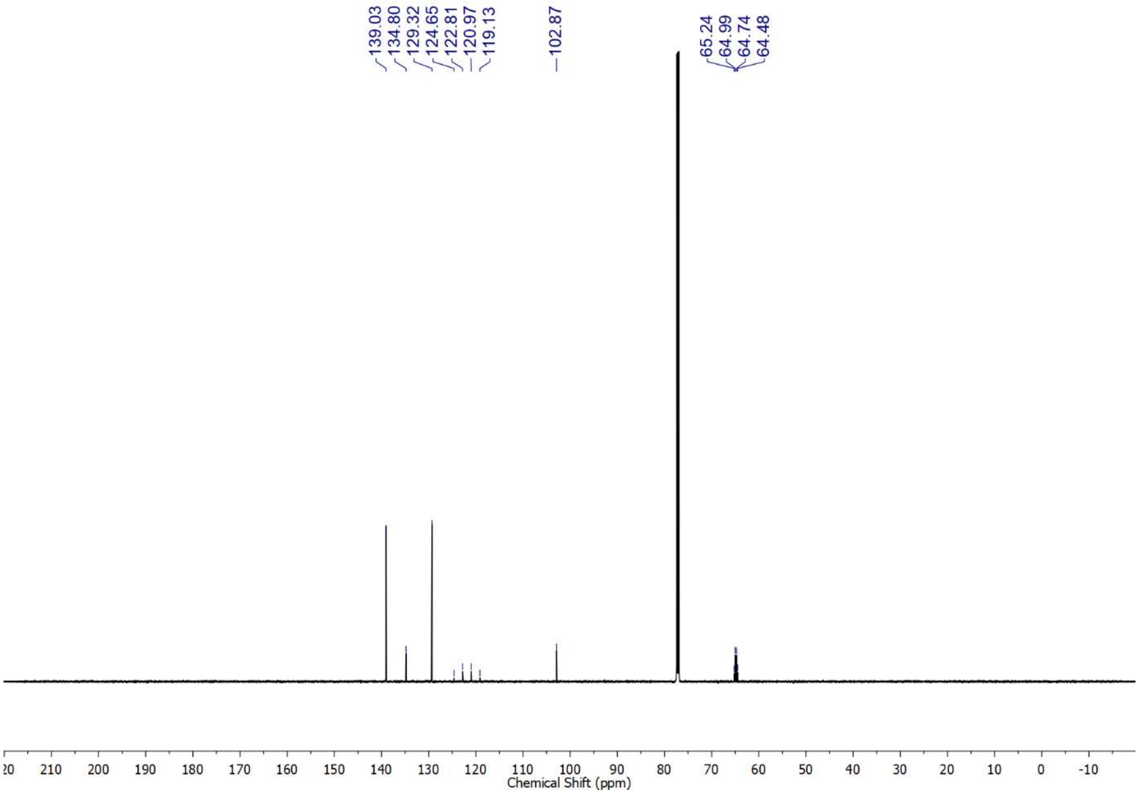

2,2,2-trifluoroethyl 4-((triisopropylsilyl)ethynyl)benzenesulfonate

^{1}H NMR (600 MHz, $CDCl_3$)

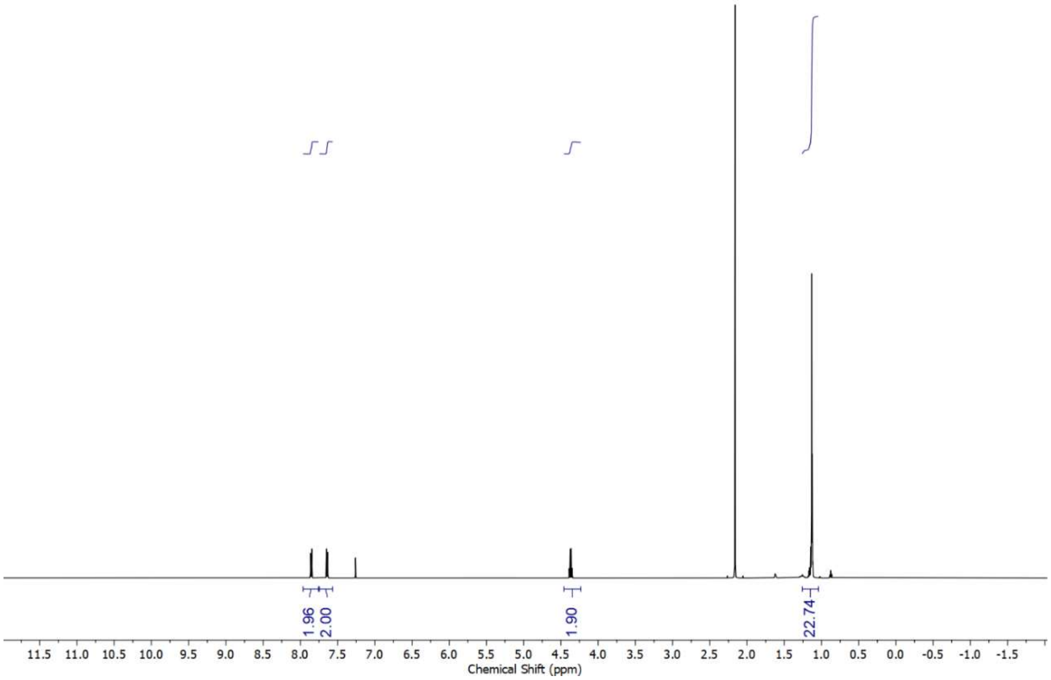

^{13}C NMR (150 MHz, $CDCl_3$)

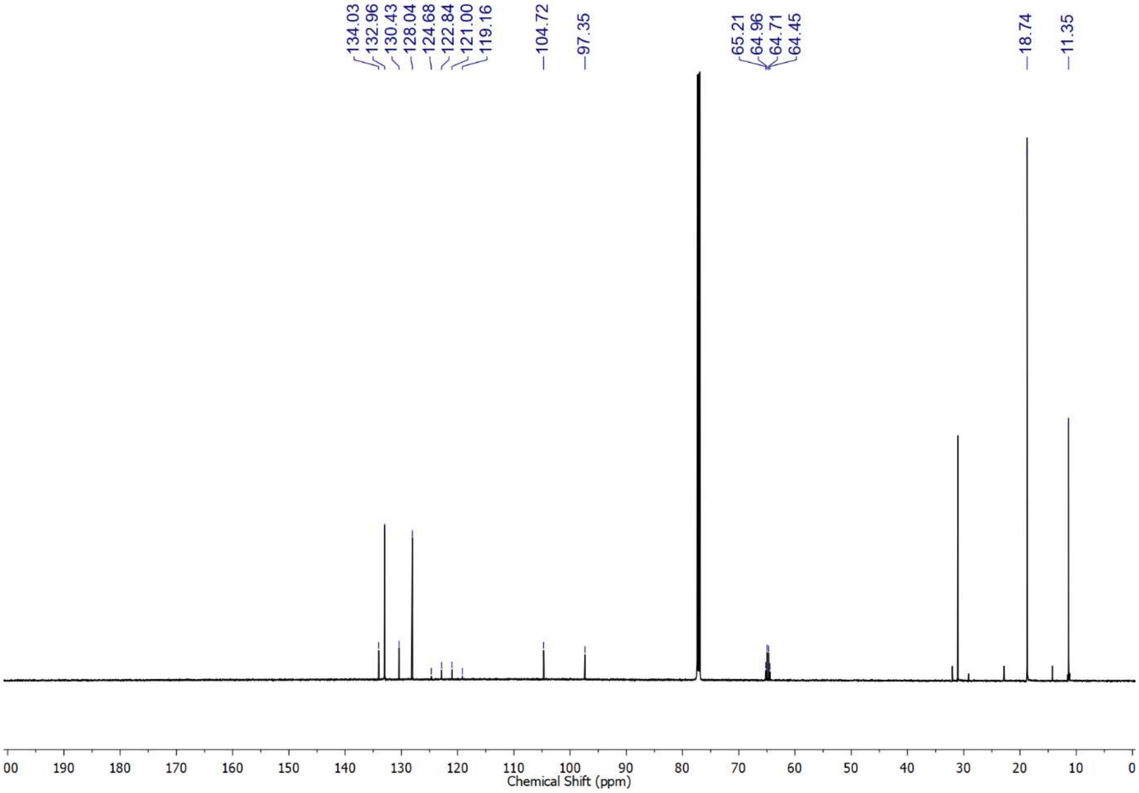

2,2,2-trifluoroethyl 4-ethynylbenzenesulfonate

^{1}H NMR (600 MHz, $CDCl_3$)

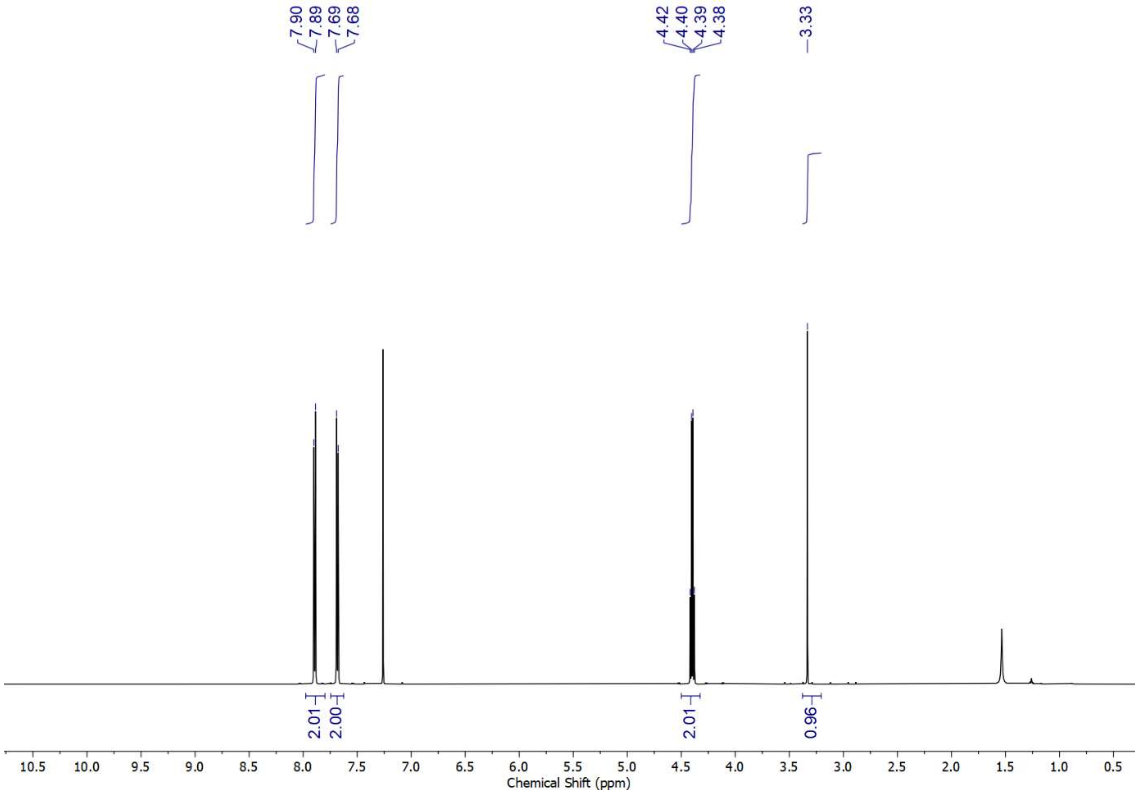

^{13}C NMR (150 MHz, $CDCl_3$)

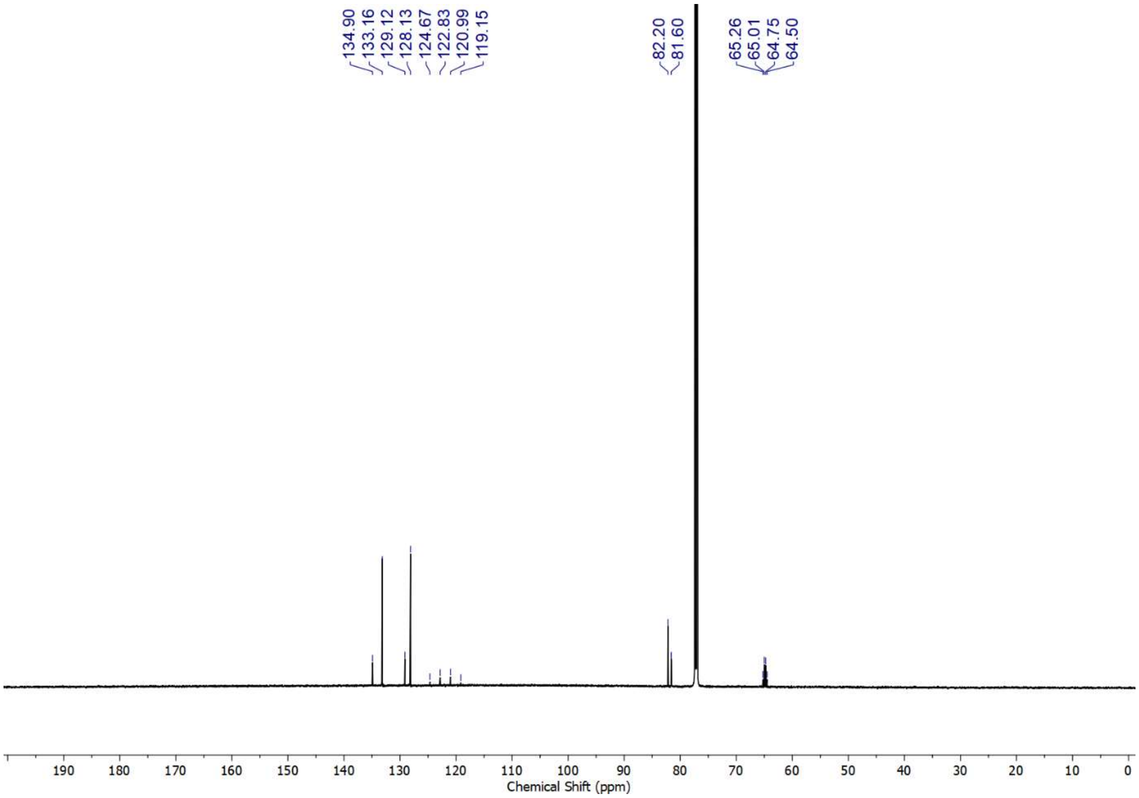

^{19}F NMR (400 MHz, $CDCl_3$)

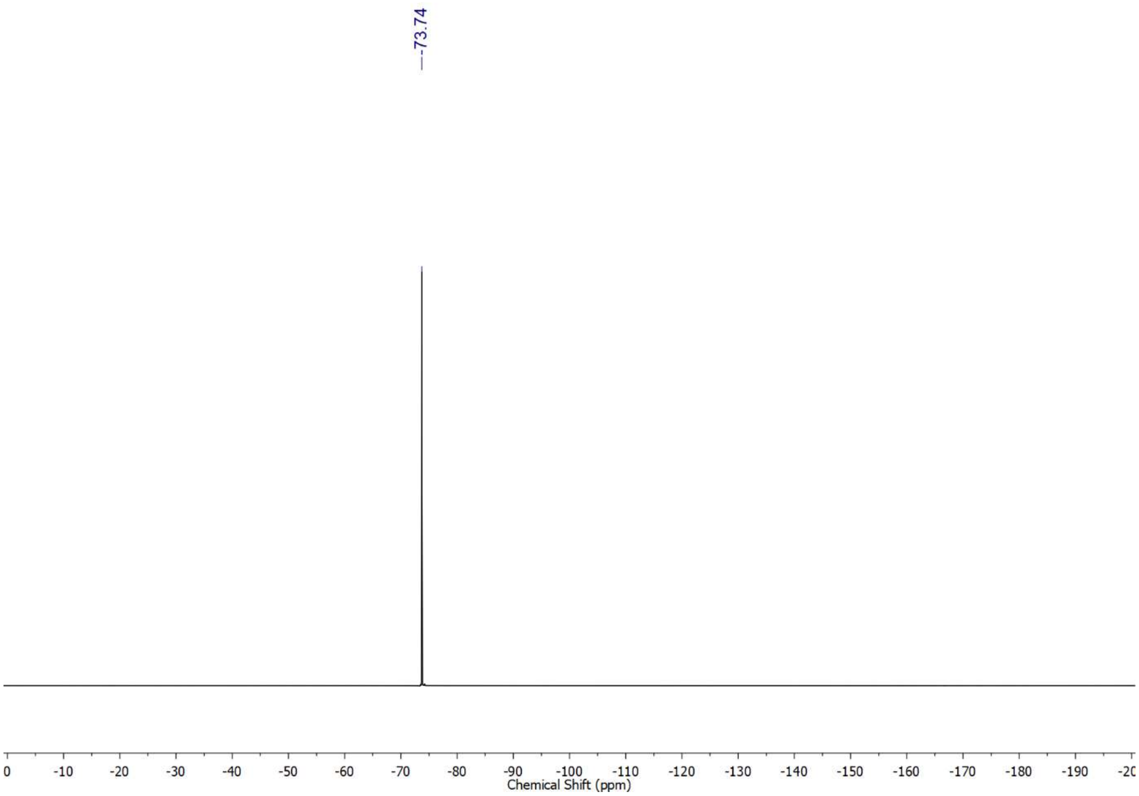

NMe_3

^{1}H NMR (600 MHz, D_2O)

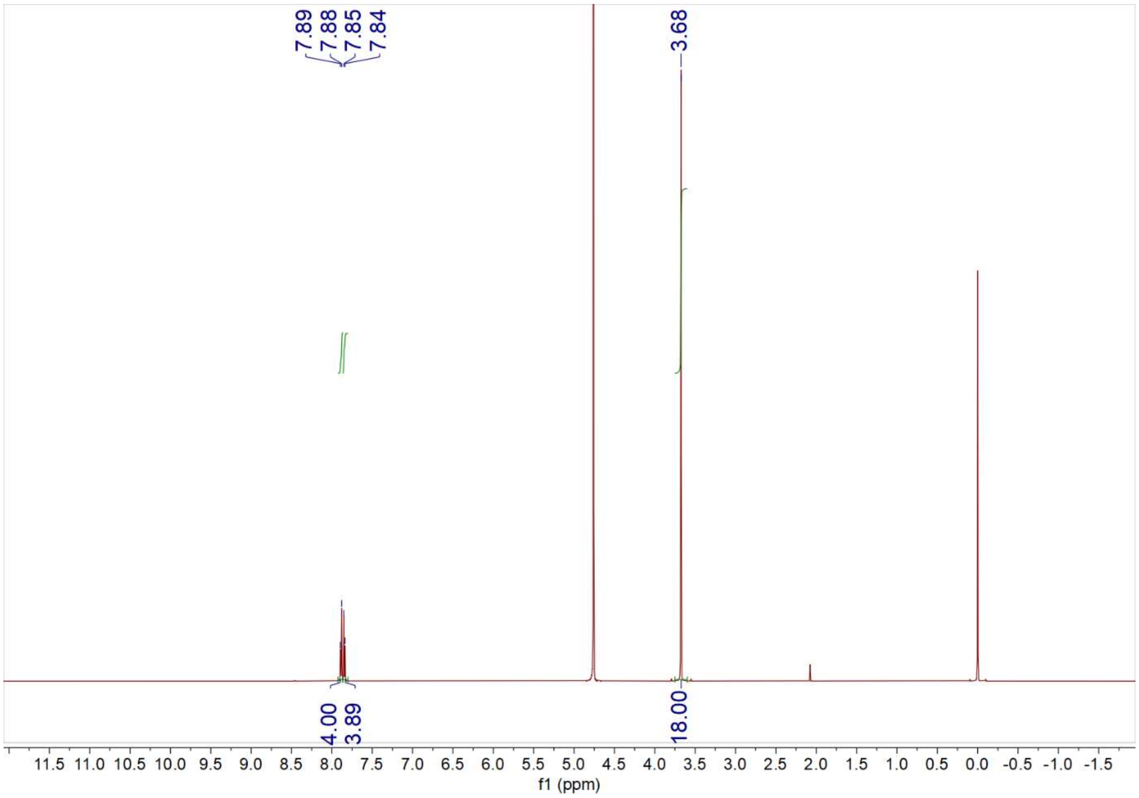

^{13}C NMR (150 MHz, D_2O)

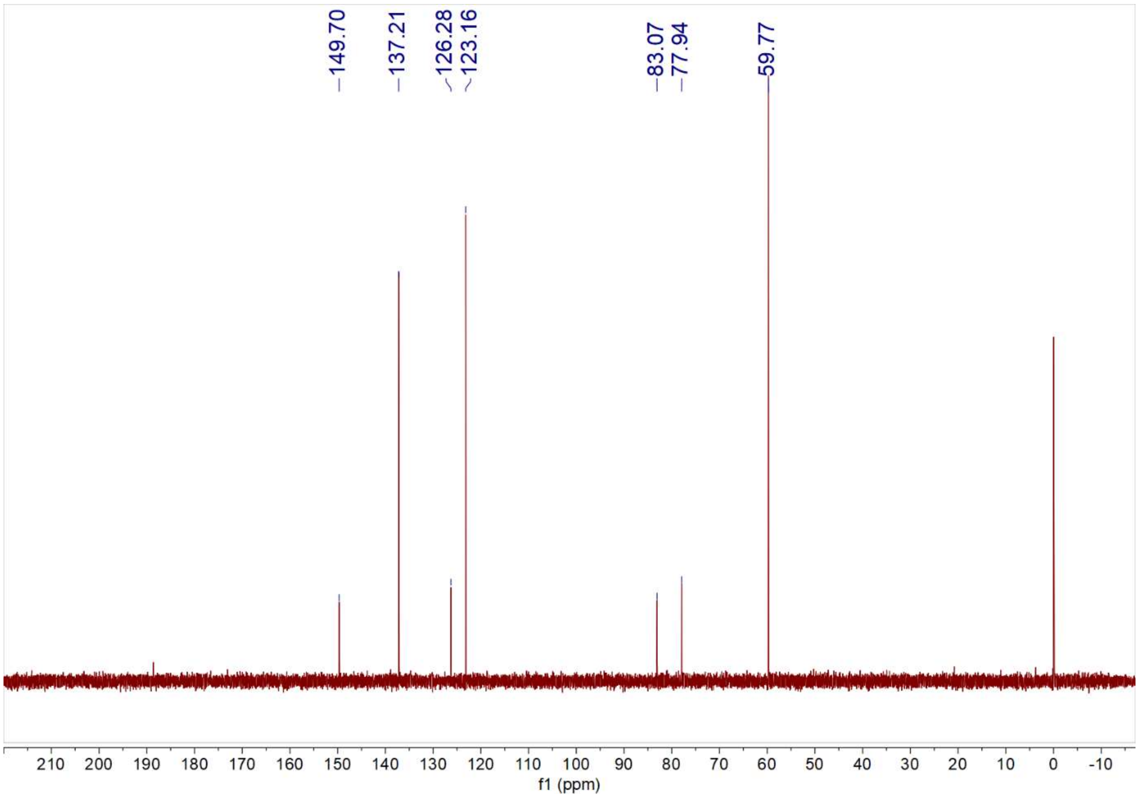

References

(1) Li, K.; Wu, K.; Lu, Y.-L.; Guo, J.; Hu, P.; Su, C.-Y. Creating Dynamic Nanospaces in Solution by Cationic Cages as Multirole Catalytic Platform for Unconventional C(Sp)−H Activation Beyond Enzyme Mimics. *Angew. Chemie - Int. Ed.* **2022**, *61*, e202114070.

(2) Xu, H.; Wu, K.; Tian, J.; Zhu, L.; Yao, X. Recyclable Cu/C3N4 Composite Catalysed Homo- and Cross-Coupling of Terminal Alkynes under Mild Conditions. *Green Chem.* **2018**, *20*, 793–797.

(3) Fasina, T. M.; Collings, J. C.; Burke, J. M.; Batsanov, A. S.; Ward, R. M.; Albesa-Jové, D.; Porrès, L.; Beeby, A.; Howard, J. A. K.; Scott, A. J.; Clegg, W.; Watt, S. W.; Viney, C.; Marder, T. B. Synthesis, Optical Properties, Crystal Structures and Phase Behaviour of Symmetric, Conjugated Ethynylarene-Based Rigid Rods with Terminal Carboxylate Groups. *J. Mater. Chem.* **2005**, *15*, 690–697.

(4) Su, L.; Dong, J.; Liu, L.; Sun, M.; Qiu, R.; Zhou, Y.; Yin, S.-F. Copper Catalysis for Selective Heterocoupling of Terminal Alkynes. *J. Am. Chem. Soc.* **2016**, *138*, 12348–12351.

(5) Vilhelmsen, M. H.; Jensen, J.; Tortzen, C. G.; Nielsen, M. B. The Glaser-Hay Reaction: Optimization and Scope Based on 13C NMR Kinetics Experiments. *European J. Org. Chem.* **2013**, No. 4, 701–711.

(6) Pati, A. K.; Mohapatra, M.; Ghosh, P.; Gharpure, S. J.; Mishra, A. K. Deciphering the Photophysical Role of Conjugated Diyne in Butadiynyl Fluorophores: Synthesis, Photophysical and Theoretical Study. *J. Phys. Chem. A* **2013**, *117*, 6548–6560.

(7) Muesmann, T. W. T.; Wickleder, M. S.; Christoffers, J. Preparation of Linear Aromatic Disulfonic Acids: New Linker Molecules for Metal-Organic Frameworks. *Synthesis (Stuttg).* **2011**, *17*, 2775–2780.

(8) Nardin, C.; Hirt, T.; Meier, W. Polymerized ABA Triblock Copolymer Vesicles. *Langmuir* **2000**, *16*, 1035–1041.
